# Molecular epidemiology and β-lactam resistance mechanisms of *Enterobacter cloacae* complex isolates obtained from bloodstream infections, Kyoto, Japan

**DOI:** 10.1128/spectrum.02485-24

**Published:** 2025-03-10

**Authors:** Akihiko Matsuo, Yasufumi Matsumura, Keiichiro Mori, Taro Noguchi, Masaki Yamamoto, Miki Nagao

**Affiliations:** 1 Department of Human Health Sciences, Graduate School of Medicine, Kyoto University 12918 , Kyoto, Japan; 2 Department of Clinical Laboratory Medicine, Graduate School of Medicine, Kyoto University 12918 , Kyoto, Japan; 3 Department of Clinical Laboratory, Kyoto University Hospital 34797 , Kyoto, Japan; Assistance Publique-Hopitaux de Paris Universite Paris Saclay, Clamart, France

**Keywords:** *Enterobacter cloacae *complex, inducible AmpC, derepressed mutants, cefotaxime, cefepime

## Abstract

The *Enterobacter cloacae* complex (ECC) comprises multiple species that require genomic analysis for precise identification. They produce inducible AmpC β-lactamase and may carry acquired β-lactamases, which are responsible for cefotaxime and cefepime resistance. To determine the molecular epidemiology, antimicrobial resistance, and β-lactam resistance mechanisms of the ECC, we conducted whole-genome sequencing analysis, antimicrobial susceptibility testing, and mutation analysis on bloodstream ECC isolates from patients in Kyoto, Japan. In 194 ECC isolates, 13 species and six unnamed taxa were identified, with *Enterobacter xiangfangensis* (36%) being the most common. A total of 38% of the isolates were nonsusceptible to cefotaxime and presented relatively high nonsusceptibility rates to all antimicrobial agents tested. Among the different species, *Enterobacter hoffmannii* presented the highest nonsusceptibility rates to both β-lactams and non-β-lactams. Among the cefotaxime-nonsusceptible isolates, 16% harbored genes encoding extended-spectrum β-lactamases (ESBLs), carbapenemase, and/or plasmid-mediated AmpC, and *ampC* derepression was the predominant resistance mechanism in the remaining isolates. The prevalent sequence types (STs) in cefotaxime-susceptible and cefotaxime-nonsusceptible isolates were different, although some STs were shared by both groups. Cefepime nonsusceptibility was detected in 7% of the isolates and was associated with *E. hoffmannii* ST78 and *E. xiangfangensis* ST93, which carry ESBLs. Sixty-four mutants, experimentally obtained from eight cefotaxime-susceptible isolates, had various *ampD* mutations, and 42% and 99% of the mutants were nonsusceptible to cefepime and piperacillin/tazobactam, respectively, indicating the risks associated with the use of these antimicrobials. Continuous surveillance via genomic and phenotypic analyses is needed to combat antimicrobial resistance in the ECC.

**IMPORTANCE:**

The *Enterobacter cloacae* complex (ECC) is a group of pathogenic bacteria that cause nosocomial infections. The ECC produces chromosomal inducible AmpC β-lactamases, which is associated with treatment failure despite initial susceptibility to third-generation cephalosporins in selected *ampC*-derepressed mutants. The complex antimicrobial resistance mechanisms of the ECC and challenges in species identification have complicated our understanding of the ECC and the selection of appropriate treatment. In this study, we performed phenotypic, whole-genome sequencing, and mutation analyses among ECC isolates from patients with bloodstream infections to determine the precise molecular-based epidemiology, resistance mechanisms to third-/fourth-generation cephalosporins, specific species and clones that contribute to antimicrobial resistance, and acquisition rates of fourth-generation cephalosporin resistance in *ampC-*derepressed mutants. These data will help elucidate the local epidemiology and complex β-lactam resistance mechanisms in the ECC and guide appropriate antimicrobial therapy and infection control strategies for ECC-related infections.

## INTRODUCTION

The *Enterobacter cloacae* complex (ECC) is a group of major pathogenic bacteria that cause nosocomial infections and is associated with respiratory tract, surgical wound, urinary tract, and bloodstream infections (1). They produce chromosomal inducible AmpC β-lactamases. The ECC is intrinsically resistant to the potent inducers ampicillin, first-generation cephalosporins, and cephamycins because of their vulnerability to AmpC, even at basal expression levels (2, 3). Third-generation cephalosporins (3GCs, i.e., cefotaxime, ceftriaxone, and ceftazidime), piperacillin-tazobactam, and aztreonam are weak inducers. Wild-type ECC is susceptible to these antimicrobial agents, although they are affected by AmpC. The minimum inhibitory concentrations (MICs) of these antimicrobial agents for the ECC can be increased if sufficient levels of AmpC are produced through induction or by *ampC*-derepressed mutants. The ECC has a high rate of spontaneous mutations that cause *ampC* derepression (4). The use of 3GCs increases the selection of *ampC*-derepressed mutants by eliminating susceptible (wild-type) subpopulations; this selection leads to acquired resistance to 3GCs during β-lactam therapy and a higher rate of treatment failure among ECC than that for other members of Enterobacterales that encode inducible AmpC (1, 2, 5–8). Isolates that are initially resistant to 3GCs usually exhibit an AmpC-hyperproducing phenotype or produce carbapenemases or extended-spectrum β-lactamases (ESBLs) (9). Therefore, 3GC treatment for ECC is discouraged even if testing shows that an isolate is susceptible to these agents (10). In contrast, carbapenems and cefepime are stable against AmpC hydrolysis and are treatment options. Clinically, cefepime allows a carbapenem-sparing therapy, and the results of several observational clinical studies support its use for the treatment of ECC infections (3, 11). However, the MIC of cefepime for *ampC*-derepressed ECC mutants can often increase above its breakpoint (12), and cefepime may not be active against ESBL producers (3, 11, 13).

The ECC comprises multiple species, and its taxonomy has been updated over time. Genome-based identification using whole-genome sequencing (WGS) data provides accurate species classification; to date, at least 24 species and 14 potential taxa have been defined using WGS (14–16). Among the genomes registered in the GenBank database, the most common human-related species is *Enterobacter xiangfangensis*, followed by *Enterobacter hoffmannii, Enterobacter asburiae*, *Enterobacter roggenkampii*, and *Enterobacter kobei* (15). The importance of accurate species identification is supported by the associations of specific species with severe disease or antimicrobial resistance. *E. xiangfangensis* and *E. hoffmannii* are associated with higher mortality, longer hospital stays, and higher rates of resistance to antimicrobials in patients with bacteremia (17). Fatal neonatal sepsis is associated with *Enterobacter bugandensis* (18). Furthermore, high-risk ECC clones with carbapenem or 3GC resistance have been identified: *E. xiangfangensis* sequence type (ST) 90, ST93, ST114, and ST171 and *E. hoffmannii* ST78 with carbapenemases (19, 20) and ST78 and ST114 with *bla*_CTX-M-15_ ESBL (9, 20, 21).

In this study, we aimed to determine the species and clonal distribution, antimicrobial susceptibility testing (AST) profiles, and cefotaxime/cefepime resistance mechanisms, including inducible/derepressed chromosomal AmpC and acquired β-lactamases, in bloodstream ECC isolates at a university hospital in Japan through a combination of phenotypic, genomic, and mutation analyses.

## RESULTS

### Species and clonal distribution

**Fig 1:**
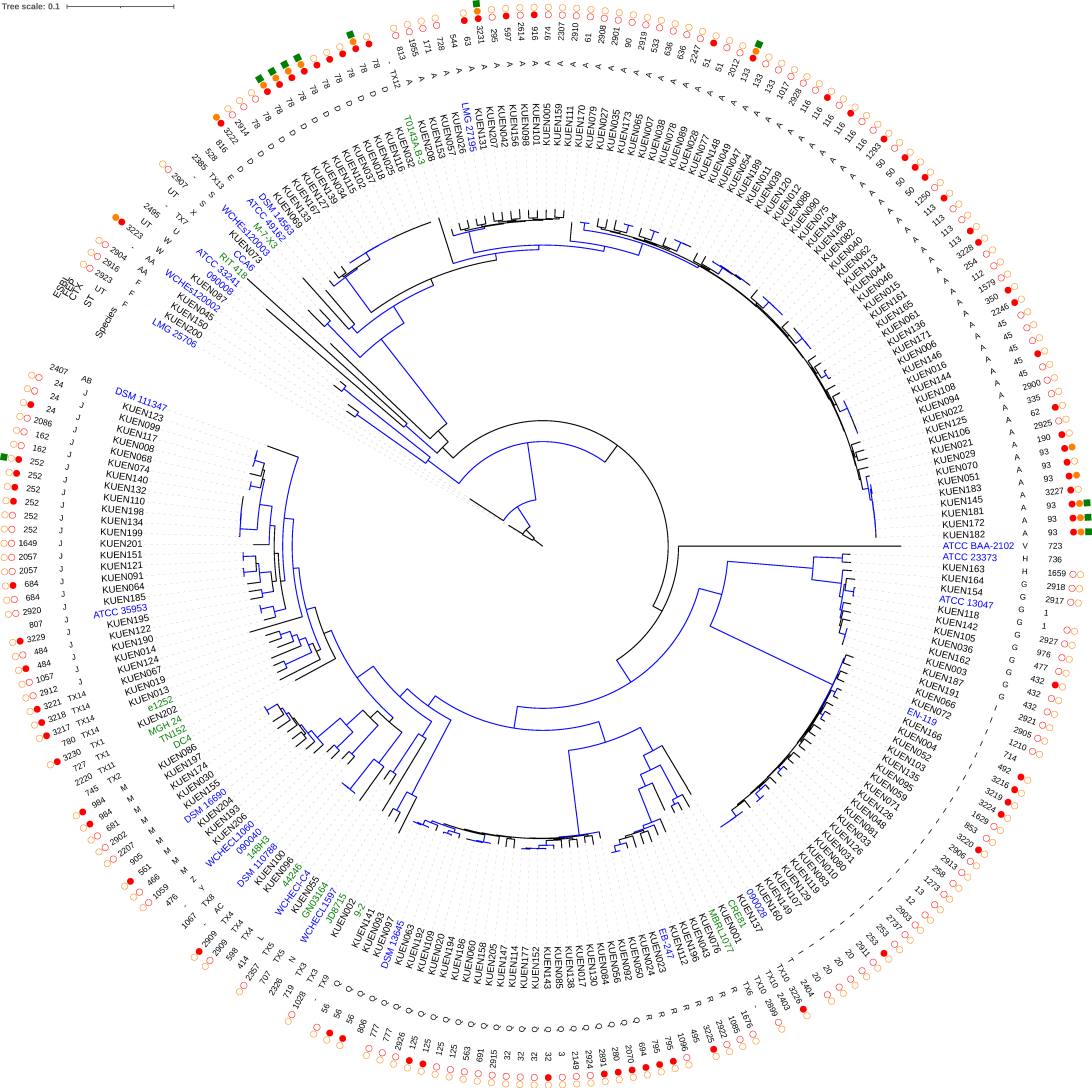
Phylogenetic tree of 194 clinical *Enterobacter cloacae* complex isolates collected in Kyoto, Japan, 2002–2018. This maximum-likelihood tree was built using 2,143 core SNPs and rooted using *Enterobacter soli* LMG 25861^T^. The tree includes 24 *Enterobacter* type strains (blue), and 14 reference strains for unnamed species (Taxon 1**–**14; green). Branches with bootstrap support of >90% from 100 replicates are highlighted in blue. In the ST column, “UT” indicates untypeable due to a lack of one or more genes, and “-” indicates unregistered. STs 2899 to 2928 and 3216 to 3231 were novel STs identified in this study. The filled circles in the CTX and FEP columns indicate nonsusceptibility to cefotaxime or cefepime, whereas the circle outlines indicate susceptibility. In the species column, the characters and species correspond as follows: A, *E. xiangfangensis*; D, *E. hoffmannii*; E, *E. hormaechei*; F, *E. mori*; G, *E. cloacae*; H, *E. dissolvens*; I, *E. ludwigii*; J, *E. asburiae*; L, *E. chengduensis*; M, *E. roggenkampii*; N, *E. sichuanensis*; Q, *E. kobei*; R, *E. bugandensis*; S, *E. quasihormaechei*; T, *E. chuandaensis*; U, *E. cancerogenus*; V, *E. soli*; W, *E. huaxiensis*; X, *E. oligotrophicus*; Y, *E. quasimori*; Z, *E. quasiroggenkampii*; AA, *E. wuhouensis*; AB, *E. dykesii*; AC, *E. vonholyi*; and TX1–14, unnamed taxon 1–14.

Among the 194 non-duplicate bloodstream ECC isolates included in the study, 120 (62%) were susceptible to cefotaxime. WGS analysis identified 13 species and six unnamed taxa in the study isolates (Fig. 1, Table 1, and Data set 1). *E. xiangfangensis* (36%), *Enterobacter ludwigii* (13%)*, E. kobei* (12%), and *E. asburiae* (12%) were the most common species, with a prevalence >10%. *E. hoffmannii* was the only species with a different prevalence in cefotaxime-nonsusceptible (CTX-NS) and cefotaxime-susceptible (CTX-S) isolates (14% vs. 3%, *P* = 0.005). The study isolates included 114 STs, 46 of which were novel STs. The most common STs in the CTX-S and CTX-NS isolates were different: *E. ludwigii* ST20 (*n* = 4), *E. xiangfangensis* ST45 and ST116 (*n* = 4 each), and *E. kobei* ST32 (*n* = 3) were prevalent in the CTX-S isolates, whereas *E. hoffmannii* ST78 (*n* = 9), *E. xiangfangensis* ST93 and ST50 (*n* = 6 and *n* = 3, respectively), and *E. asburiae* ST252 (*n* = 4) were prevalent in the CTX-NS isolates. The CTX-S and CTX-NS isolates shared 16 STs, namely, *E. hoffmannii* ST78, *E. xiangfangensis* ST116, *E. asburiae* ST252, *E. xiangfangensis* ST45, *E. kobei* ST32, *E. xiangfangensis* ST50, *E. xiangfangensis* ST113, *E. kobei* ST125, *E. asburiae* ST24, *E. kobei* ST56, *E. xiangfangensis* ST133, *E. cloacae* ST432, *E. xiangfangensis* ST51, *E. ludwigii* ST253, *E. asburiae* ST484, and *E. asburiae* ST684 (in decreasing order by number of isolates), which corresponded to 26% and 45% of the CTX-S and CTX-NS isolates, respectively. Among these 16 STs, the phylogenetic analysis (Fig. 1) indicated that isolates belonging to the same ST were monophyletic or CTX-NS isolates clustered within the same branch as CTX-S isolates, except one isolate, CTX-NS ST113, which was placed in a different branch. CTX-NS isolates from ST78, ST252, and ST133 carried ESBLs.

**TABLE 1:** Species distribution, antimicrobial nonsusceptibility, and β-lactamase genes of clinical *Enterobacter cloacae* complex isolates obtained from blood cultures at Kyoto University Hospital in Kyoto, Japan, 2002–2018

	N (%)		
Variables	Total (*n* = 194)	Cefotaxime-susceptible (*n* = 120)	Cefotaxime-nonsusceptible (*n* = 74)
Species			
*E. xiangfangensis*	69 (36%)	42 (35%)	27 (36%)
*E. ludwigii*	25 (13%)	19 (16%)	6 (8%)
*E. kobei*	24 (12%)	15 (13%)	9 (12%)
*E. asburiae*	23 (12%)	15 (13%)	8 (11%)
*E. hoffmannii* ^*a*^	13 (7%)	3 (3%)	10 (14%)
*E. cloacae*	9 (5%)	8 (7%)	1 (1%)
*E. roggenkampii*	8 (4%)	5 (4%)	3 (4%)
*E. bugandensis*	7 (4%)	4 (3%)	3 (4%)
Taxon 14	3 (2%)	0	3 (4%)
*E. mori*	2 (1%)	2 (2%)	0
Taxon 4	2 (1%)	1 (1%)	1 (1%)
Taxon 10	2 (1%)	1 (1%)	1 (1%)
*E. dissolvens*	1 (1%)	1 (1%)	0
*E. huaxiensis*	1 (1%)	0 (0%)	1 (1%)
*E. quasihormaechei*	1 (1%)	1 (1%)	0
*E. wuhouensis*	1 (1%)	1 (1%)	0
Taxon 1	1 (1%)	0	1 (1%)
Taxon 3	1 (1%)	1 (1%)	0
Taxon 5	1 (1%)	1 (1%)	0
Antimicrobial nonsusceptibility			
Cefoxitin	193 (99%)	119 (99%)	74 (100%)
Cefotaxime^*a*^	74 (38%)	0	74 (100%)
Ceftazidime^*a*^	55 (28%)	0	55 (74%)
Cefepime^*a*^	14 (7%)	0	14 (19%)
Piperacillin^*a*^	71 (37%)	13 (11%)	58 (78%)
Piperacillin-tazobactam^*a*^	43 (22%)	0	43 (58%)
Aztreonam^*a*^	48 (25%)	0	48 (65%)
Imipenem	0	0	0
Meropenem	1 (1%)	0	1 (1%)
Ciprofloxacin^*a*^	31 (16%)	9 (8%)	22 (30%)
Levofloxacin^*a*^	27 (14%)	6 (5%)	21 (28%)
Gentamicin^*a*^	4 (2%)	0	4 (5%)
Tobramycin^*a*^	8 (4%)	1 (1%)	7 (9%)
Amikacin	3 (2%)	0	3 (4%)
Minocycline^*a*^	19 (10%)	7 (6%)	12 (16%)
Sulfamethoxazole-trimethoprim^*a*^	31 (16%)	14 (12%)	17 (23%)
Colistin	67 (35%)	39 (33%)	28 (38%)
β-Lactamase gene			
Chromosomal *ampC*			
*bla*_ACT_	175 (90%)	105 (88%)	70 (95%)
*bla*_CMH_	10 (5%)	9 (8%)	1 (1%)
*bla*_MIR_	8 (4%)	5 (4%)	3 (4%)
Carbapenemases			
*bla*_GES-24_	1 (1%)	0	1 (1%)
*bla*_IMP-1_	1 (1%)	0	1 (1%)
Extended-spectrum β-lactamase gene			
*bla*_CTX-M-3_^*a*^	9 (5%)	0	9 (12%)
*bla*_SHV-12_	3 (2%)	0	3 (4%)
Plasmid-mediated *ampC*			
*bla*_DHA-1_	1 (1%)	0	1 (1%)
Broad-spectrum β-lactamase gene			
*bla*_LAP-2_	4 (2%)	1 (1%)	3 (4%)
*bla*_TEM-1_	17 (9%)	11 (9%)	6 (8%)

^*a*^ *P* value <0.05 for cefotaxime-susceptible vs. cefotaxime-nonsusceptible isolates.

### AST and antimicrobial resistance genes

The CTX-NS isolates had higher nonsusceptibility rates than the CTX-S isolates for all antimicrobial agents tested, although the differences in the rates varied (Table 1). All CTX-S isolates were susceptible to ceftazidime, cefepime, piperacillin/tazobactam, aztreonam, imipenem, meropenem, gentamicin, and amikacin. Significant differences in the nonsusceptibility rates were observed for ceftazidime, cefepime, piperacillin, piperacillin/tazobactam, aztreonam, ciprofloxacin, levofloxacin, gentamicin, tobramycin, minocycline, and sulfamethoxazole-trimethoprim. All the isolates carried chromosomal AmpC genes that were characteristic of their respective species (Table 1): *bla*_CMH_ in *E. cloacae* and *Enterobacter dissolvens*, *bla*_MIR_ in *E. roggenkampii*, and *bla*_ACT_ in all the other species. ESBL, carbapenemase, and plasmid-mediated AmpC genes were found in 15%, 3%, and 1% of the CTX-NS isolates, respectively (16% overall, with overlap). The most common ESBL gene was *bla*_CTX-M-3_, followed by *bla*_SHV-12_. Among the 14 cefepime-nonsusceptible isolates, 11 were resistant (MICs > 8 µg/mL), and three were susceptible-dose dependent (MICs 4–8 µg/mL). Nine cefepime-resistant isolates carried ESBL genes. The remaining resistant isolate and the three susceptible dose-dependent isolates were negative for any β-lactamase genes except broad-spectrum β-lactamase genes. ST78 and ST93 were predominant in these cefepime-nonsusceptible isolates (frequently with ESBLs; Fig. 1). The distribution of antimicrobial resistance genes other than those included in Table 1 is shown in Table S1. Among 44 genes, seven genes, *aac(6')-Iaj*, *aadA1*, *dfrA15*, *dfrA19*, *fosA*, *qacEΔ1*, and *sul1*, were more frequently carried by CTX-NS isolates than by CTX-S isolates.

**Fig 2:**
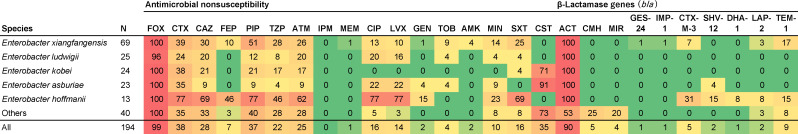
Prevalence of antimicrobial nonsusceptibility and β-lactamase genes among major *Enterobacter cloacae* complex species. Five species found in >10 isolates are shown. This heatmap displays rates along a gradient from green (0%) to red (100%). Among the nonmajor species, *E. cloacae* (89%), *E. roggenkampii* (88%), *E. bugandensis* (86%), Taxon 14 (100%), Taxon 4 (100%), *E. dissolvens* (100%), and Taxon 1 (100%) presented high colistin nonsusceptibility rates. FOX, cefoxitin; CTX, cefotaxime; CAZ, ceftazidime; FEP, cefepime; PIP, piperacillin; TZP, piperacillin-tazobactam; ATM, aztreonam; IPM, imipenem; MEM, meropenem; CIP, ciprofloxacin; LVX, levofloxacin; GEN, gentamicin; TOB, tobramycin; AMK, amikacin; MIN, minocycline; SXT, sulfamethoxazole-trimethoprim; CST, colistin.

Fig. 2 compares the AST and β-lactamase genes of the major ECC species (*n* > 10). *E. hoffmannii* had the highest nonsusceptibility rate for cefotaxime, ceftazidime, cefepime, piperacillin, piperacillin/tazobactam, aztreonam, ciprofloxacin, levofloxacin, gentamicin, minocycline, and sulfamethoxazole-trimethoprim. The ESBL genes *bla*_CTX-M-3_ and *bla*_SHV-12_ were most prevalent in *E. hoffmannii. E. asburiae* had the highest nonsusceptibility rate for colistin, followed by *E. kobei* and species other than the major species.

Among the 120 CTX-S isolates, except one isolate that was susceptible to cefoxitin, the addition of 8 µg/mL cefoxitin antagonized cefotaxime (100%), ceftazidime (100%), piperacillin/tazobactam (24%), and cefepime (6%) but did not antagonize ciprofloxacin, levofloxacin, imipenem, and meropenem (Fig. S1).

To determine the changes in the above microbiological characteristics over time, we compared the isolates from 2002–2010 to those from 2011–2018 (Table S2). The species distribution did not differ significantly; however, the prevalence of ST32, the tobramycin nonsusceptibility rate, and the carriage of *bla*_CTX-M-3_ significantly decreased after 2010. Three ST93 isolates and one ST133 isolate, both of which were nonsusceptible to tobramycin and carried *bla*_CTX-M-3_, were detected only in 2002–2010. A greater than 25% decrease in nonsusceptibility rates was observed for cefepime, fluoroquinolones, aminoglycosides, minocycline, and sulfamethoxazole-trimethoprim, although these differences did not reach statistical significance.

### Optimal cefoxitin concentration for AmpC induction and detection of inducible AmpC

We determined the optimal cefoxitin concentration to be 8 µg/mL and inducible AmpC β-lactamase activity (please refer to Supplementary Results and Discussion for details).

### Mutation analysis

**Fig 3:**
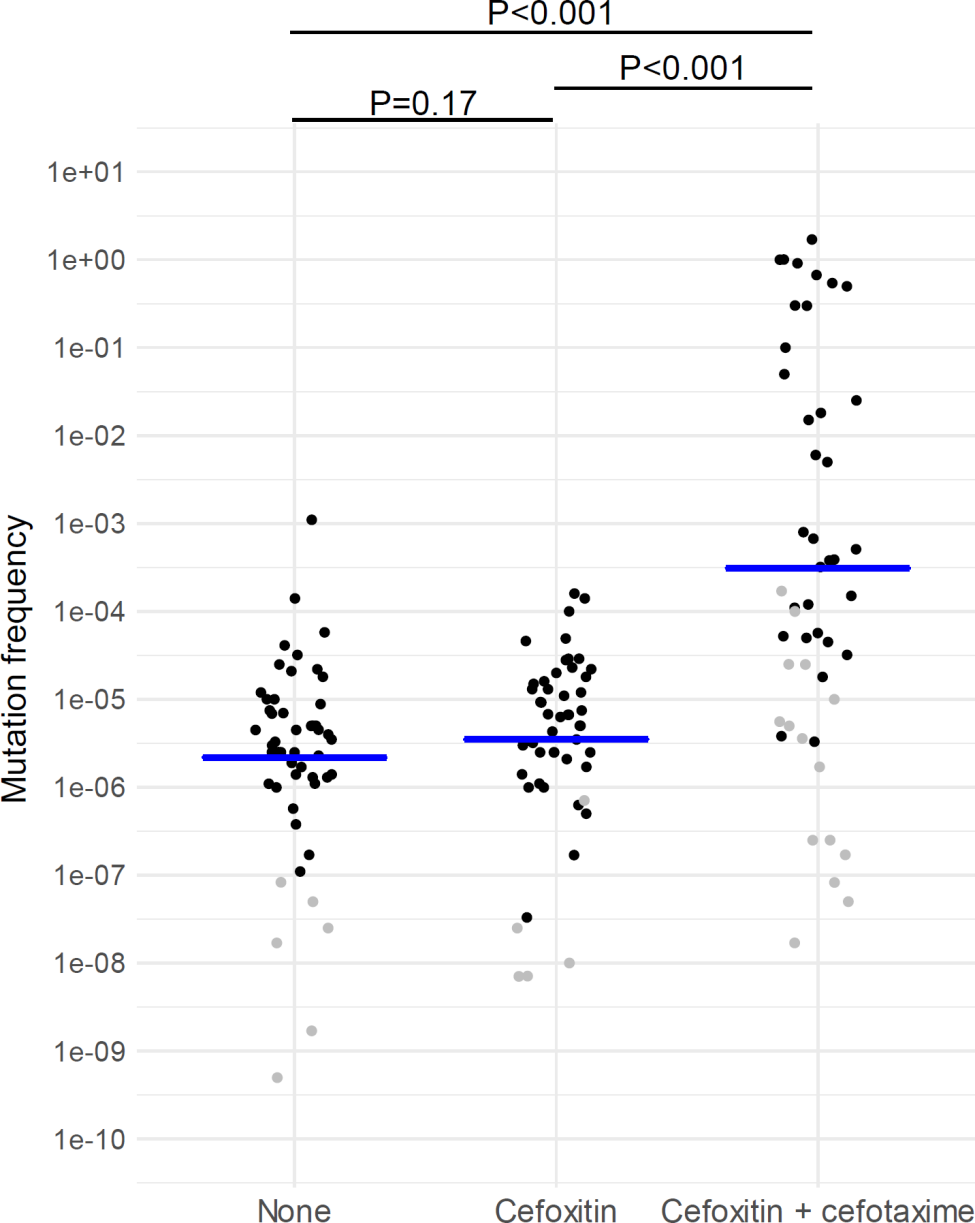
Frequencies of *ampC* derepressed mutants among 50 cefotaxime-susceptible *Enterobacter cloacae* complex isolates**.** The blue bars indicate the geometric means. The black circles indicate the presence of the mutants, whereas the gray circles indicate their absence. The limit of detection, which was calculated based on the assumption that only one mutant was present, was recorded when the mutant was absent. Data are not shown for the two isolates that did not grow in cultures with cefoxitin and cefotaxime. Statistical comparison was performed for the isolates that produced mutants.

The identification of *ampC*-derepressed mutants was performed in the 50 randomly selected CTX-S (subset 1) isolates (Table S6). These mutants were frequently identified in cultures with no antimicrobial agent (88%), and those with cefoxitin (90%), and the mean mutation frequencies were similar (4.5 × 10^−6^ vs. 6.1 × 10^−6^; *P* = 0.17; Fig. 3). The mutants occurred less frequently in cultures with cefoxitin and cefotaxime (66%, *P* = 0.03 compared with cultures with no antimicrobial agent); however, the mutation frequencies were significantly higher than those of cultures with no antimicrobial agent or with cefoxitin (3.2 × 10^−3^; *P* < 0.001 each). Figs. S5 and S6 show the distributions of mutation frequencies and ratios according to species.

**Fig 4:**
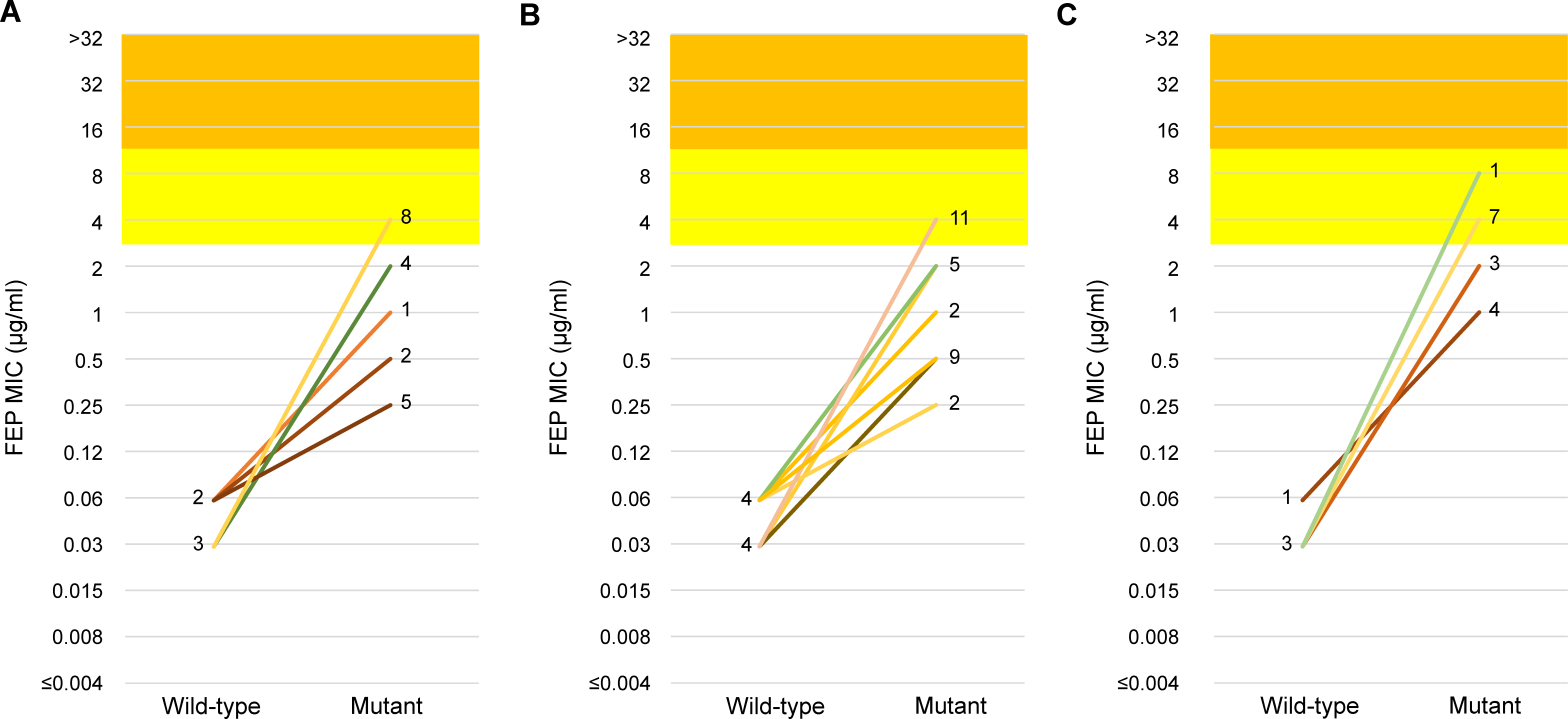
Comparison of the MICs of cefepime between the wild-type isolates and their *ampC* derepressed mutants. Mutants obtained from the cultures without antimicrobials, those with cefoxitin, and those with cefoxitin and cefotaxime, were shown in panels A, B, and C, respectively. The number of wild-type isolates or mutants with each MIC value is shown next to the connecting lines. The susceptible, susceptible dose-dependent, and resistant categories are indicated by white, yellow, and orange backgrounds.

A total of 64 mutants were obtained from eight arbitrarily selected isolates (a maximum of four mutants per culture condition) for identification of the genetic mutations (Data set 1). Four, three, and one mutants were obtained from 15, 1, and 1 isolate-culture conditions, respectively (Data set 1). All the mutants had *ampD* mutations (Table 2), including missense mutations (*n* = 22), frameshift mutations (*n* = 20), deletion or truncation mutations (*n* = 14), and nonsense mutations (*n* = 8). Two or more mutation types were identified in mutants from the same isolate in cultures without antimicrobial agents (5/5, 100%), cultures with cefoxitin (3/7, 43% excluding the isolate with only one mutant), and cultures with cefoxitin and cefotaxime (1/4, 25%). Different *ampD* mutants were found in the same isolate under different culture conditions (4/5, 80%), except for one isolate that had the same T233G mutation in cultures without antimicrobial agents and with cefoxitin and cefotaxime. Non-silent mutations in genes other than *ampD* were found in seven mutants (11%), and silent mutations or mutations in noncoding regions were found in nine mutants (14%), six of which (9%) had both types of mutations (Data set 2). The non-silent mutations included deletions in the *ampD-ampE* region (*n* = 2), *nuoM* (NADH-quinone oxidoreductase subunit M; *n* = 1), and *paaZ* (bifunctional protein, *n* = 1) and missense mutations in *mpaA* (murein peptide amidase A; *n* = 1) and *intS* (prophage integrase; *n* = 1). All the mutants were nonsusceptible to cefotaxime, ceftazidime, aztreonam, and piperacillin. The MICs of cefepime increased by a median of 64-fold (range: 4–128), and 27 mutants (42%) were nonsusceptible (categorized as susceptible, dose-dependent; Fig. 4; Data set 2 ), accounting for 40%, 38%, and 53% of the mutants obtained from the cultures with no antimicrobial agents, cefoxitin, and cefoxitin and cefotaxime, respectively. One or more cefepime-nonsusceptible mutants were obtained from 60% (3/5), 38% (3/8), and 75% (3/4) of the isolates. The MICs for piperacillin/tazobactam increased by a median of 16-fold (range: 4 to ≥64), and 63 mutants (99%) were nonsusceptible. Susceptibilities to other antimicrobial agents were unchanged.

**TABLE 2:** Nucleotide changes in the *ampD* gene of cefotaxime-resistant mutant strains in comparison with their wild-type strains^*a*^

	Antimicrobials used in mutation experiments (number of mutants with mutations)		
Strain	None	Cefoxitin	Cefoxitin and cefotaxime
KUEN003	A109C (S37R, *n* = 2), G65A (R22H, *n* = 1), C493T (P165S, *n* = 1)	Δ280–281 (frameshift, *n* = 4)	Δ364–564 by *IS*2-like insertion (truncation, *n* = 4)
KUEN021	ND	T233A (I78N, *n* = 4)	ND
KUEN022	ND	G478T (F160Stop, *n* = 1)	ND
KUEN028	T233G (I78S, *n* = 3), G496 (G166R, *n* = 1)	T233A (I78N, *n* = 2), 345delT (frameshift, *n* = 2)	T233G (I78S, *n* = 2), G340T (G114Stop, *n* = 1)
KUEN042	G352T (E118Stop, *n* = 3), C223T (H75Y, *n* = 1)	A491C (D164A, *n* = 2), 164insA (frameshift, *n* = 1), G278C (R93P, *n* = 1)	511delT (frameshift, *n* = 4)
KUEN048	163delA (frameshift, *n* = 1), 169delA (frameshift, *n* = 1), 370insC (frameshift, *n* = 1), Δ1–564^*b*^ (deletion, *n* = 1)	Δ97–564^*c*^ (truncation, *n* = 1), T233A (I78N, *n* = 1), C261A (Y87Stop, *n* = 1), T419G (L140R, *n* = 1)	ND
KUEN049	G21A (W7Stop, *n* = 1), 135delT (frameshift, *n* = 1), G285A (W95Stop, *n* = 1), Δ394–402 (frameshift, *n* = 1)	Δ249–564^*d*^ (truncation, *n* = 4)	314delG (frameshift, *n* = 1)
KUEN076	ND	Δ186–552^*e*^ (truncation, *n* = 4)	ND

^*a*^ ND, not detected.

^*b*^ The *orf*-*ampE*-*ampD*-*nadC* region (3,440 bp) was deleted.

^*c*^ Nucleotide (nt) 97 was fused to nt 28 of the adjacent *ampE* gene. The parent strain had 9 common nucleotides (CTGCTGGTT) at *ampD* nt 88–96 and *ampE* nt 19–27.

^*d*^ Nucleotide 249 was fused to nt 332 of the adjacent *ampE* gene. The parent strain had 8 common nucleotides (GATGGCGA) at *ampD* nt 241–248 and *ampE* nt 324–331.

^*e*^ Nucleotide 186 was fused to an intergenic region.

## DISCUSSION

### Species identification

Accurate species identification is important for determining the clinical and microbiological characteristics of the ECC, a group comprising multiple bacterial species. In this context, WGS-based analysis is required instead of conventional identification methods based on biochemical properties, mass spectrometry, or *hsp65* (22). Species distribution data from clinical isolates are currently limited because WGS-based testing methods are difficult to implement in clinical laboratory practices. The results of previous WGS-based studies agree with our determination that *E. xiangfangensis* is the most common species (17, 23–27), taking into account the recent nomenclature changes (15). The second and third most common species varied among the studies: *E. hoffmannii* and *E. bugandensis* (China, bloodstream infections, 2016–2018) (17), *E. kobei* and *E. roggenkampii* (China, clinical isolates, 2019–2020) (23), *E. bugandensis* and *E. asburiae* (Guadeloupe, clinical isolates, 2018) (24), *E. asburiae* and *E. kobei* (Japan, IMP-1-producing clinical isolates, 2007–2011) (25), *E. kobei* and *E. roggenkampii* (Japan, bloodstream infections, 2017–2019) (26), and *E. asburiae* and *E. kobei*/*E. ludwigii* (Japan, clinical isolates, 2017–2018) (27). *E. ludwigii* was common in our study (13%) and in two previous Japanese studies (10%–16%, except the study for carbapenemase-producing isolates) (26, 27), but its identification was rare (≤2%) in the other three studies from countries other than Japan, suggesting regional differences in species distribution.

In this study, *E. hoffmannii* was found to be nonsusceptible to multiple antimicrobial agents, including β-lactams, fluoroquinolone, and sulfamethoxazole-trimethoprim, which is consistent with a previous study reporting that *E. xiangfangensis* and *E. hoffmannii* exhibited similar resistance characteristics (17). Several species, such as *E. asburiae* and *E. kobei*, were associated with colistin nonsusceptibility in this study. A Japanese single-center study of clinical ECC isolates reported high rates of colistin resistance in *E. roggenkampii*, *E. kobei*, *Enterobacter chuandaensis*, *E. cloacae*, and *E. dissolvens* (27).

### CTX-NS rates and resistance mechanisms

Previously reported CTX-NS (or 3GC-resistant) rates in clinical ECC isolates ranged from 29% to 58% (17, 23, 24, 28), which is consistent with the CTX-NS rate (38%) reported in this study. ESBLs (15% prevalence) were the most common acquired β-lactamases responsible for cefotaxime-nonsusceptibility, and cefoxitin-resistant isolates without these β-lactamases (84%) are considered to have an *ampC*-derepressed phenotype (29). Several surveillance studies have reported the 3GC resistance mechanisms of the ECC. Among 652 ECC isolates with ceftazidime resistance and increased MICs of cefepime from 77 U.S. medical centers from 2017 to 2019, the acquired β-lactamases ESBLs, carbapenemases, and plasmid-mediated AmpC accounted for 15%, 6%, and 1%, respectively, of all isolates, and the remaining 78% of isolates were classified as *ampC*-derepressed mutants without these β-lactamases (28). Izdebski et al. investigated 195 3GC-resistant ECC isolates from 12 hospitals across Europe and Israel from 2008 to 2011 and reported that both the *ampC*-derepressed phenotype and ESBL or carbapenemase genes were present in 52% of isolates, with 3% of the isolates having both characteristics (9). ESBL genes were found in 49% of the isolates. In this multilocus sequence typing (MLST)-based analysis, the *ampC*-derepressed isolates were highly polyclonal; in contrast, isolates carrying *bla*_CTX-M-15_ were associated with sporadic clonal spread. None of the prevalent, widespread clones of ST78, ST66, ST114, and ST108 had unique ESBL or carbapenemase profiles, and all of these STs also included *ampC*-derepressed isolates without these β-lactamases (9). Similarly, in our study, ST78 and ST93 were mainly associated with *bla*_CTX-M-3_, but they also included *ampC*-derepressed isolates without ESBLs or carbapenemases (Fig. 1 and Data set 1). These observations suggest that the sporadic spread of CTX-NS ECC clones that independently acquired ESBLs has contributed to increased CTX-NS ECC isolates in Japan and other countries. The prevalent STs differed between the CTX-S and CTX-NS isolates; however, some prevalent (e.g., ST78, ST116, ST252, ST45, ST32, and ST50) and non-prevalent STs without ESBLs were common to both groups, suggesting sporadic development of *ampC*-derepressed mutants and limited subsequent spread.

The prevalence of ESBL genes in ECC isolates has been reported to be 10%–58%, and *bla*_SHV_ and *bla*_CTX-M_ (particularly *bla*_SHV-12_ and *bla*_CTX-M-15_) are the most prevalent genes worldwide (30). For example, *bla*_SHV-12_ was the most common gene in a U.S. study (28), and *bla*_CTX-M-15_ was the most common gene in a European study (9). ST78 and ST114 have been associated with *bla*_CTX-M-15_ (9, 20, 21). In addition to *bla*_CTX-M-15_, *bla*_CTX-M-3_ has been reported in China, Spain, Italy, Poland, Romania, and Taiwan (30). In studies from Japan and China, the most prevalent ESBL gene was *bla*_CTX-M-3_, followed by *bla*_SHV-12_ (23, 31), which is consistent with our results. These ESBL genes were associated with ST78 (both genes) and ST93 (*bla*_CTX-M-3_). These STs have been identified as high-risk clones associated with carbapenemase and/or ESBLs (9, 19, 20). Carbapenemases were rarely found in the study isolates, reflecting the local epidemiology of Japan (32).

### Temporal changes

An increase in multidrug-resistant ECC has been observed (33); however, there have been no reports indicating an increase in derepressed mutants. Our data indicated that the species/clonal distribution did not dramatically change over time and that antimicrobial nonsusceptibility rates, including cefepime nonsusceptibility and carriage of the ESBL gene *bla*_CTX-M-3_, exhibited decreasing trends, suggesting the absence of an ongoing spread of antimicrobial resistance in the study isolates. There are no clear explanations for these trends; we did not change the treatment strategies for ECC bacteremia or detect any outbreaks during the study period.

### Mutation experiments

In bacterial culture, *ampC*-derepressed mutants have a mean mutation rate of 2.7 × 10^−8^, as determined with a Luria–Delbrück fluctuation assay employing 16 parallel cultures of 40 clinical ECC isolates (4). The mutant and wild-type populations change due to the development and subsequent growth of mutants with a specific mutation rate. This change can be assessed with mutation frequencies, which are calculated from the proportion of mutants in bacterial cultures. Mutation frequencies are considered inaccurate and nonreproducible measures of mutation rates (4), but they may reflect the fitness costs of mutations and the selection of mutants under specific conditions. The frequency of spontaneous *ampC* derepression mutations has been reported as 10^−6^ to 10^−7^ (34). Our results revealed that the mean mutation frequencies in cultures without any antimicrobial agent and those with an inducer (cefoxitin) were similar to the reported frequencies. Increased mutation frequencies and reduced genetic variation diversity in cultures with levels of cefotaxime and cefoxitin above the MICs (where antagonism occurred) confirmed the selection of *ampC*-derepressed mutants under these culture conditions.

In our study, 42% of mutants became nonsusceptible to cefepime (all were susceptible, dose-dependent mutants with an MIC of 4–8 μg/mL), and 38%–75% produced at least one mutant that was not susceptible to cefepime. When the EUCAST breakpoints (MIC ≥2 µg/mL for nonsusceptibility) were applied, 61% of the mutants were classified as nonsusceptible, which is consistent with the recently reported value of 66% (12). Derepressed mutants have been believed to retain susceptibility to cefepime, but the data supporting this conclusion are based on the previous breakpoint (MIC 16 µg/mL for nonsusceptibility) (12, 35). Other mechanisms of cefepime resistance include specific AmpC variants that increase cefepime hydrolysis or porin deficiency, but these alterations were not found in our mutants, indicating that *ampC* derepression alone can result in cefepime nonsusceptibility. Experimental studies indicate that a high dose of cefepime is needed to suppress the development of *ampC*-derepressed mutants (36). Furthermore, an inoculum effect was detected with cefepime, which compromised the effectiveness against severe or high-inoculum infections (37). Clinical data are conflicting, but one study suggests that cefepime treatment for ECC bloodstream infections caused by cefepime-susceptible-dose dependent isolates is associated with increased mortality (38). Considering these data, cefepime may not be the treatment of choice for CTX-S ECC, especially for severe infections and when co-administered with potent AmpC inducers. Similarly, piperacillin/tazobactam may not be the optimal choice, as recommended by the guidelines (39), considering that most mutants identified in this study were nonsusceptible to piperacillin/tazobactam, that the ability of tazobactam to inhibit AmpC is limited, and that observational studies have suggested poorer outcomes.

Mutations responsible for *ampC* derepression are most frequently found in *ampD*, followed by *ampR* and *ampG* (3, 24, 40, 41). Genome-wide comparisons between our CTX-S isolates and the corresponding mutants revealed multiple different *ampD* mutations in all the mutants, with other mutations (neither *ampR* nor *ampG*) with unknown effects found in 11% of mutants. No mutants had porin or efflux mutations.

This study has several limitations. First, the number of study isolates was relatively small to fully capture ECC diversity, resistance patterns, the characteristics of each species, or their trends. The included isolates were not collected from all clinical specimen types, and those selected for the subsets were potentially biased. Second, the study only covered one institution in Japan, potentially limiting the generalizability of the results. Third, we could not assess the 3GC resistance mechanisms of CTX-NS isolates (and mutants), including AmpC activity and mutations contributing to the derepressed status. Fourth, mutations in porin and efflux pumps were not investigated. However, these mutations may not be very important for β-lactam resistance in the presence of chromosomal AmpC. The associations of carbapenem resistance with porin and/or efflux mutations combined with AmpC are well known (42); in contrast, in the presence of AmpC, resistance to 3GCs can occur regardless of outer membrane permeability (43). CTX-NS mechanisms of all *ampC* derepressed mutants can be explained by *ampD* mutations, and none of the mutants developed porin or efflux mutations. Fifth, mutation experiments were limited to specific concentrations and antimicrobial agents. Mutant analysis of sub-MIC levels of exposure to β-lactams, especially cefepime, is needed to clarify the risk of development or selection of *ampC*-derepressed mutants. Mutants might be missed because of restrictions on culture conditions (high limit of detection for mutation frequencies in several isolate-culture conditions; Fig. 3) and the lack of replicate experiments.

In conclusion, the current molecular epidemiology and β-lactam resistance mechanisms were investigated in bloodstream ECC isolates in Kyoto, Japan. The major findings include the species distribution, AST profiles, distribution of antimicrobial resistance genes, specific species associated with antimicrobial resistance and specific clones carrying ESBL genes that contribute to cefepime nonsusceptibility, and the frequent development of cefepime and piperacillin/tazobactam-nonsusceptible *ampC*-derepressed mutants from CTX-S isolates. Our data will help elucidate the local epidemiology and complex β-lactam resistance mechanisms in the ECC and guide appropriate antimicrobial therapy and infection control strategies for ECC infections. Continuous genomic and phenotypic studies using isolates collected from a wider range of institutions with clinical data are needed to combat antimicrobial resistance in the ECC.

## MATERIALS AND METHODS

### Bacterial strains

Non-duplicate CTX-S clinical ECC isolates obtained from blood cultures at Kyoto University Hospital in Kyoto, Japan, from February 2002 to December 2018 were included. The bacterial strains were identified by matrix-assisted laser desorption ionization-time of flight mass spectrometry using MBT compass software (version 4.1; Bruker Daltonics, Bremen, Germany).

### AST

Antimicrobial susceptibility was evaluated by broth microdilution using customized frozen plates (Frozen Plate Eiken, Eiken Chemical, Tokyo, Japan) according to the 2022 Clinical and Laboratory Standards Institute (CLSI) guidelines (44). Susceptible, dose-dependent isolates were classified as nonsusceptible. Three sets of 96-well plates were used to test cefotaxime, ceftazidime, piperacillin/tazobactam, cefepime, levofloxacin, ciprofloxacin, imipenem, meropenem, cefoxitin, piperacillin, aztreonam, gentamicin, tobramycin, amikacin, minocycline, trimethoprim-sulfamethoxazole, and colistin. For the CTX-S isolates, the first eight antimicrobials were also tested with 8 µg/mL cefoxitin, and a more than 2-fold increase in the MIC with the addition of cefoxitin was considered antagonistic. *Escherichia coli* ATCC 25922 was used as a quality control for susceptibility testing.

### Optimal cefoxitin concentration for AmpC induction and detection of inducible AmpC

The methods used for these experiments are described in Supplementary Methods.

### Detection of *ampC*-derepressed mutants

After the bacterial suspension was prepared and incubated at 35°C for 18 h according to the CLSI guidelines for AST (44), serial dilutions of the cultures obtained from the wells with no antimicrobials, 8 µg/mL cefoxitin, and 8 µg/mL cefoxitin and 4 µg/mL cefotaxime were inoculated on Mueller‒Hinton agar with or without 8 µg/mL cefotaxime and incubated for 18 h. Bacterial colony counts were performed, and the mutation frequency was calculated by dividing the number of colonies from cultures with 8 µg/mL cefotaxime by the number of colonies from cultures without antimicrobial agents. One 90-mm culture plate was used for each isolate-culture condition.

### WGS

We used the Illumina DNA Prep kit (Illumina, San Diego, CA, USA) to prepare libraries for sequencing. The samples were multiplexed and sequenced on an Illumina NovaSeq 6000 or NextSeq 1000 for 300 cycles (159 bp paired-end).

### Genomic analysis

Draft genomes were obtained using SPAdes version 3.15.4 and annotated using Prokka v1.14.5. Species were identified according to average nucleotide identity (ANI) with a cutoff of 95% against the 24 type strains and 14 reference strains for unnamed species (Data set 1) (15, 45). ANI values based on BLAST were calculated with JSpecies (16). To define the presence of genes and their alleles, we used the following databases or typing schemes: AMRFinderPlus (46) and MLST (http://pubmlst.org/ecloacae/). We created a core SNP-based phylogenetic tree using kSNP 3.0 (47). The tree was visualized using iTOL v6 (https://itol.embl.de/). To identify differences between the wild-type and mutant pairs, reads obtained from the mutants were mapped to a draft genome of a wild-type strain using bwa version 0.7.18. Variants were called using BCFtools version 1.20 if the depth of coverage was ≥10 and the quality score was ≥20. *ampD* mutations were identified by comparison of draft genomes using BLASTn version 2.12.0.

### Statistical analysis

Continuous variables were analyzed with a 2-tailed paired Mann–Whitney U test. Categorical variables were compared using Fisher’s exact test. Statistical analyses were performed using R software version 4.1.2 (https://cran.r-project.org).

## Data Availability

WGS read data were deposited in the NCBI SRA database under accession number PRJNA1167922. Other data obtained in this study are available within the manuscript and supplemental materials.

## SUPPLEMENTAL MATERIAL

Supplemental data spectrum.02485-24-s0001.xlsx Data sets S1 and S2.

Supplemental material spectrum.02485-24-s0002.docx Tables S1 to S6; Fig. S1 to S6.

ASM does not own the copyrights to Supplemental Material that may be linked to, or accessed through, an article. The authors have granted ASM a non-exclusive, world-wide license to publish the Supplemental Material files. Please contact the corresponding author directly for reuse.

## References

[1] Sanders WE, Sanders CC. 1997. Enterobacter spp.: pathogens poised to flourish at the turn of the century. Clin Microbiol Rev 10:220–241. doi:10.1128/CMR.10.2.220 9105752 PMC172917

[2] Jacoby GA. 2009. AmpC beta-lactamases. Clin Microbiol Rev 22:161–182. doi:10.1128/CMR.00036-08 19136439 PMC2620637

[3] Tamma PD, Doi Y, Bonomo RA, Johnson JK, Simner PJ, Antibacterial Resistance Leadership Group. 2019. A primer on AmpC β-lactamases: necessary knowledge for an increasingly multidrug-resistant world. Clin Infect Dis 69:1446–1455. doi:10.1093/cid/ciz173 30838380 PMC6763639

[4] Kohlmann R, Bähr T, Gatermann SG. 2018. Species-specific mutation rates for ampC derepression in Enterobacterales with chromosomally encoded inducible AmpC β-lactamase. J Antimicrob Chemother 73:1530–1536. doi:10.1093/jac/dky084 29566147

[5] Muller A, Lopez-Lozano JM, Bertrand X, Talon D. 2004. Relationship between ceftriaxone use and resistance to third-generation cephalosporins among clinical strains of Enterobacter cloacae. J Antimicrob Chemother 54:173–177. doi:10.1093/jac/dkh282 15150164

[6] Chow JW, Fine MJ, Shlaes DM, Quinn JP, Hooper DC, Johnson MP, Ramphal R, Wagener MM, Miyashiro DK, Yu VL. 1991. Enterobacter bacteremia: clinical features and emergence of antibiotic resistance during therapy. Ann Intern Med 115:585–590. doi:10.7326/0003-4819-115-8-585 1892329

[7] Nicolle LE. 1988. Prior antimicrobial therapy and resistance of Enterobacter, Citrobacter and Serratia to third generation cephalosporins. J Hosp Infect 11:321–327. doi:10.1016/0195-6701(88)90084-9 2899583

[8] Choi SH, Lee JE, Park SJ, Choi SH, Lee SO, Jeong JY, Kim MN, Woo JH, Kim YS. 2008. Emergence of antibiotic resistance during therapy for infections caused by Enterobacteriaceae producing AmpC beta-lactamase: implications for antibiotic use. Antimicrob Agents Chemother 52:995–1000. doi:10.1128/AAC.01083-07 18086837 PMC2258504

[9] Izdebski R, Baraniak A, Herda M, Fiett J, Bonten MJM, Carmeli Y, Goossens H, Hryniewicz W, Brun-Buisson C, Gniadkowski M, MOSAR WP2, WP3 and WP5 Study Groups. 2015. MLST reveals potentially high-risk international clones of Enterobacter cloacae. J Antimicrob Chemother 70:48–56. doi:10.1093/jac/dku359 25216820

[10] Leclercq R, Cantón R, Brown DF, Giske CG, Heisig P, MacGowan AP, Mouton JW, Nordmann P, Rodloff AC, Rossolini GM, Soussy CJ, Steinbakk M, Winstanley TG, Kahlmeter G. 2013. EUCAST expert rules in antimicrobial susceptibility testing. Clin Microbiol Infect 19:141–160. doi:10.1111/j.1469-0691.2011.03703.x 22117544

[11] Meini S, Tascini C, Cei M, Sozio E, Rossolini GM. 2019. AmpC β-lactamase-producing Enterobacterales: what a clinician should know. Infection 47:363–375. doi:10.1007/s15010-019-01291-9 30840201

[12] Kohlmann R, Bähr T, Gatermann SG. 2019. Effect of ampC derepression on cefepime MIC in Enterobacterales with chromosomally encoded inducible AmpC β-lactamase. Clin Microbiol Infect 25:1158. doi:10.1016/j.cmi.2019.05.007 31128286

[13] Tamma PD, Aitken SL, Bonomo RA, Mathers AJ, van Duin D, Clancy CJ. 2022. Infectious diseases society of America 2022 guidance on the treatment of extended-spectrum beta-lactamase producing Enterobacterales (ESBL-E), carbapenem-resistant Enterobacterales (CRE), and Pseudomonas aeruginosa with difficult-to-treat resistance (DTR-P. aeruginosa). Clin Infect Dis 75:187–212. doi:10.1093/cid/ciac268doi:35439291 PMC9890506

[14] Chen Y, Xiang G, Liu P, Zhou X, Guo P, Wu Z, Yang J, Chen P, Huang J, Liao K. 2024. Prevalence and molecular characteristics of ceftazidime-avibactam resistance among carbapenem-resistant Pseudomonas aeruginosa clinical isolates. J Glob Antimicrob Resist 36:276–283. doi:10.1016/j.jgar.2024.01.014 38295902

[15] Wu W, Feng Y, Zong Z. 2020. Precise species identification for Enterobacter: a genome sequence-based study with reporting of two novel species, Enterobacter quasiroggenkampii sp nov. and Enterobacter quasimori sp. mSystems 5:e00527-20. doi:10.1128/mSystems.00527-20 32753511 PMC7406230

[16] Richter M, Rosselló-Móra R. 2009. Shifting the genomic gold standard for the prokaryotic species definition. Proc Natl Acad Sci U S A 106:19126–19131. doi:10.1073/pnas.0906412106 19855009 PMC2776425

[17] Wu W, Wei L, Feng Y, Xie Y, Zong Z. 2021. Precise species identification by whole-genome sequencing of Enterobacter bloodstream infection, China. Emerg Infect Dis 27:161–169. doi:10.3201/eid2701.190154 33350909 PMC7774573

[18] Girlich D, Ouzani S, Emeraud C, Gauthier L, Bonnin RA, Le Sache N, Mokhtari M, Langlois I, Begasse C, Arangia N, Fournier S, Fortineau N, Naas T, Dortet L. 2021. Uncovering the novel Enterobacter cloacae complex species responsible for septic shock deaths in newborns: a cohort study. Lancet Microbe 2:e536–e544. doi:10.1016/S2666-5247(21)00098-7 35544179

[19] Peirano G, Matsumura Y, Adams MD, Bradford P, Motyl M, Chen L, Kreiswirth BN, Pitout JDD. 2018. Genomic epidemiology of global carbapenemase-producing Enterobacter spp., 2008-2014. Emerg Infect Dis 24:1010–1019. doi:10.3201/eid2406.171648 29774858 PMC6004858

[20] Gomez-Simmonds A, Annavajhala MK, Wang Z, Macesic N, Hu Y, Giddins MJ, O’Malley A, Toussaint NC, Whittier S, Torres VJ, Uhlemann A-C. 2018. Genomic and geographic context for the evolution of high-risk carbapenem-resistant Enterobacter cloacae complex clones ST171 and ST78. MBio 9:e00542-18. doi:10.1128/mBio.00542-18 29844109 PMC5974468

[21] Girlich D, Poirel L, Nordmann P. 2015. Clonal distribution of multidrug-resistant Enterobacter cloacae*.* Diagn Microbiol Infect Dis 81:264–268. doi:10.1016/j.diagmicrobio.2015.01.003 25680336

[22] Davin-Regli A, Lavigne JP, Pagès JM. 2019. Enterobacter spp.: update on taxonomy, clinical aspects, and emerging antimicrobial resistance. Clin Microbiol Rev 32:e00002-19. doi:10.1128/CMR.00002-19 31315895 PMC6750132

[23] Dong X, Zhu M, Li Y, Huang D, Wang L, Yan C, Zhang L, Dong F, Lu J, Lin X, Li K, Bao Q, Cong C, Pan W. 2022. Whole-genome sequencing-based species classification, multilocus sequence typing, and antimicrobial resistance mechanism analysis of the Enterobacter cloacae complex in southern China. Microbiol Spectr 10:e0216022. doi:10.1128/spectrum.02160-22 36350178 PMC9769718

[24] Pot M, Reynaud Y, Couvin D, Ducat C, Ferdinand S, Gravey F, Gruel G, Guérin F, Malpote E, Breurec S, Talarmin A, Guyomard-Rabenirina S. 2021. Wide distribution and specific resistance pattern to third-generation cephalosporins of Enterobacter cloacae complex members in humans and in the environment in Guadeloupe (French West Indies). Front Microbiol 12:628058. doi:10.3389/fmicb.2021.628058 34248862 PMC8268024

[25] Aoki K, Harada S, Yahara K, Ishii Y, Motooka D, Nakamura S, Akeda Y, Iida T, Tomono K, Iwata S, Moriya K, Tateda K. 2018. Molecular characterization of IMP-1-producing Enterobacter cloacae complex isolates in Tokyo. Antimicrob Agents Chemother 62:e02091-17. doi:10.1128/AAC.02091-17 29311089 PMC5826111

[26] Sarangi J, Matsuo N, Nonogaki R, Hayashi M, Kawamura K, Suzuki M, Jin W, Tamai K, Ogawa M, Wachino JI, Kimura K, Yagi T, Arakawa Y. 2022. Molecular epidemiology of Enterobacter cloacae complex isolates with reduced carbapenem susceptibility recovered by blood culture. Jpn J Infect Dis 75:41–48. doi:10.7883/yoken.JJID.2021.141 34193664

[27] Fukuzawa S, Sato T, Aoki K, Yamamoto S, Ogasawara N, Nakajima C, Suzuki Y, Horiuchi M, Takahashi S, Yokota SI. 2023. High prevalence of colistin heteroresistance in specific species and lineages of Enterobacter cloacae complex derived from human clinical specimens. Ann Clin Microbiol Antimicrob 22:60. doi:10.1186/s12941-023-00610-1 37454128 PMC10350281

[28] Sader HS, Mendes RE, Doyle TB, Davis AP, Castanheira M. 2021. Characterization of Enterobacter cloacae and Citrobacter freundii species complex isolates with decreased susceptibility to cephalosporins from United States hospitals and activity of ceftazidime/avibactam and comparator agents. JAC Antimicrob Resist 3:dlab136. doi:10.1093/jacamr/dlab136 34430873 PMC8378278

[29] Jeong SH, Song W, Park MJ, Kim JS, Kim HS, Bae IK, Lee KM. 2008. Boronic acid disk tests for identification of extended-spectrum beta-lactamase production in clinical isolates of Enterobacteriaceae producing chromosomal AmpC beta-lactamases. Int J Antimicrob Agents 31:467–471. doi:10.1016/j.ijantimicag.2007.12.014 18337065

[30] Yeh TK, Lin HJ, Liu PY, Wang JH, Hsueh PR. 2022. Antibiotic resistance in Enterobacter hormaechei. Int J Antimicrob Agents 60:106650. doi:10.1016/j.ijantimicag.2022.106650 35934231

[31] Kanamori H, Yano H, Hirakata Y, Hirotani A, Arai K, Endo S, Ichimura S, Ogawa M, Shimojima M, Aoyagi T, Hatta M, Yamada M, Gu Y, Tokuda K, Kunishima H, Kitagawa M, Kaku M. 2012. Molecular characteristics of extended-spectrum beta-lactamases and qnr determinants in Enterobacter species from Japan. PLoS ONE 7:e37967. doi:10.1371/journal.pone.0037967 22719857 PMC3376121

[32] Kayama S, Yahara K, Sugawara Y, Kawakami S, Kondo K, Zuo H, Kutsuno S, Kitamura N, Hirabayashi A, Kajihara T, Kurosu H, Yu L, Suzuki M, Hisatsune J, Sugai M. 2023. National genomic surveillance integrating standardized quantitative susceptibility testing clarifies antimicrobial resistance in Enterobacterales. Nat Commun 14:8046. doi:10.1038/s41467-023-43516-4 38052776 PMC10698200

[33] Annavajhala MK, Gomez-Simmonds A, Uhlemann AC. 2019. Multidrug-reesistant Enterobacter cloacae complex emerging as a global, diversifying threat. Front Microbiol 10:44. doi:10.3389/fmicb.2019.00044 30766518 PMC6365427

[34] Livermore DM. 1987. Clinical significance of beta-lactamase induction and stable derepression in gram-negative rods. Eur J Clin Microbiol 6:439–445. doi:10.1007/BF02013107 3311738

[35] Fung-Tomc JC, Gradelski E, Huczko E, Dougherty TJ, Kessler RE, Bonner DP. 1996. Differences in the resistant variants of Enterobacter cloacae selected by extended-spectrum cephalosporins. Antimicrob Agents Chemother 40:1289–1293. doi:10.1128/AAC.40.5.1289 8723487 PMC163312

[36] Negri MC, Baquero F. 1999. In vitro selective concentrations of cefepime and ceftazidime for AmpC beta-lactamase hyperproducer Enterobacter cloacae variants. Clin Microbiol Infect 5 Suppl 1:S25–S28. doi:10.1111/j.1469-0691.1999.tb00721.x 11869274

[37] Thomson KS, Moland ES. 2001. Cefepime, piperacillin-tazobactam, and the inoculum effect in tests with extended-spectrum beta-lactamase-producing Enterobacteriaceae. Antimicrob Agents Chemother 45:3548–3554. doi:10.1128/AAC.45.12.3548-3554.2001 11709338 PMC90867

[38] Lee NY, Lee CC, Li CW, Li MC, Chen PL, Chang CM, Ko WC. 2015. Cefepime therapy for monomicrobial Enterobacter cloacae bacteremia: unfavorable outcomes in patients infected by cefepime-susceptible dose-dependent isolates. Antimicrob Agents Chemother 59:7558–7563. doi:10.1128/AAC.01477-15 26416853 PMC4649147

[39] Tamma PD, Heil EL, Justo JA, Mathers AJ, Satlin MJ, Bonomo RA. 2024. Infectious diseases society of America 2024 guidance on the treatment of antimicrobial-resistant gram-negative infections. Clin Infect Dis:ciae403. doi:10.1093/cid/ciae403 39108079

[40] Babouee Flury B, Ellington MJ, Hopkins KL, Turton JF, Doumith M, Woodford N. 2016. The differential importance of mutations within AmpD in cephalosporin resistance of Enterobacter aerogenes and Enterobacter cloacae. Int J Antimicrob Agents 48:555–558. doi:10.1016/j.ijantimicag.2016.07.021 27665520

[41] Babouee Flury B, Ellington MJ, Hopkins KL, Turton JF, Doumith M, Woodford N, Loy R, Staves P, Hinic V, Frei R, Woodford N. 2016. Association of novel nonsynonymous single nucleotide polymorphisms in ampD with cephalosporin resistance and phylogenetic variations in ampC, ampR, ompF, and ompC in Enterobacter cloacae isolates that are highly resistant to carbapenems. Antimicrob Agents Chemother 60:2383–2390. doi:10.1128/AAC.02835-15 26856839 PMC4808197

[42] Davin-Regli A, Pagès JM, Vergalli J. 2024. The contribution of porins to Enterobacterial drug resistance. J Antimicrob Chemother 79:2460–2470. doi:10.1093/jac/dkae265 39205648

[43] Masi M, Vergalli J, Ghai I, Barba-Bon A, Schembri T, Nau WM, Lafitte D, Winterhalter M, Pagès JM. 2022. Cephalosporin translocation across Enterobacterial OmpF and OmpC channels, a filter across the outer membrane. Commun Biol 5:1059. doi:10.1038/s42003-022-04035-y 36198902 PMC9534850

[44] CLSI. 2022. Performance standards for antimicrobial susceptibility testing. In CLSI supplement M100, 32nd ed. Clinical and Laboratory Standards Institute, Wayne, PA.

[45] Cho GS, Stein M, Fiedler G, Igbinosa EO, Koll LP, Brinks E, Rathje J, Neve H, Franz C. 2021. Polyphasic study of antibiotic-resistant Enterobacteria isolated from fresh produce in Germany and description of Enterobacter vonholyi sp. Syst Appl Microbiol 44:126174. doi:10.1016/j.syapm.2020.126174 33370657

[46] Feldgarden M, Brover V, Gonzalez-Escalona N, Frye JG, Haendiges J, Haft DH, Hoffmann M, Pettengill JB, Prasad AB, Tillman GE, Tyson GH, Klimke W. 2021. AMR finderplus and the reference gene catalog facilitate examination of the genomic links among antimicrobial resistance, stress response, and virulence. Sci Rep 11:12728. doi:10.1038/s41598-021-91456-0 34135355 PMC8208984

[47] Gardner SN, Slezak T, Hall BG. 2015. kSNP3.0: SNP detection and phylogenetic analysis of genomes without genome alignment or reference genome. Bioinformatics 31:2877–2878. doi:10.1093/bioinformatics/btv271 25913206

