# Supplementary material for: Molecular epidemiology and β-lactam resistance mechanisms of Enterobacter cloacae complex isolates obtained from bloodstream infections, Kyoto, Japan
Source: Microbiol Spectr. 2025 Mar 10;13(4):e02485-24. doi: 10.1128/spectrum.02485-24 (PMC11960451; doi:10.1128/spectrum.02485-24)
Supplement: Supplemental material — Tables S1 to S6; Fig. S1 to S6. [file spectrum.02485-24-s0002.docx]

**Supplementary material**

**Supplementary Results**

**Selection of CTX-S isolates**

From the 120 CTX-S isolates, two nested subsets were created via random selection: 50 isolates for AmpC induction and mutation experiments (subset 1; Dataset 1) and 30 isolates for determination of the optimal cefoxitin concentration for AmpC induction (subset 2).

**Optimal cefoxitin concentration for AmpC induction**

Thirty subset 2 isolates were used to determine the optimal cefoxitin concentration for AmpC induction. Disk diffusion tests supplemented with different concentrations of cefoxitin revealed that the zones of inhibition of cefotaxime and ceftazidime were significantly reduced when the cefoxitin concentration was increased from 0 to 4 µg/ml and from 4 to 8 µg/ml (Fig. S2). We selected 3 representative isolates from each of the 3 groups (A, B, and C; subset 3; Dataset 1) with cefotaxime/ceftazidime zones of inhibition of different diameters (susceptibility before induction) and changes in diameter after AmpC induction with 8 µg/ml cefoxitin. Group A showed a smaller diameter and/or smaller diameter change, group B showed a moderate diameter and/or moderate change, and group C showed a larger diameter and/or larger change (Tables S3 and S4). In all the isolates in the 3 groups, the changes in the zone of inhibition diameter for cefotaxime and ceftazidime were reversed by the addition of an AmpC inhibitor (cloxacillin) (Dataset 1; Tables S3 and S4), and bacterial growth was not inhibited in the presence of 8 µg/ml cefoxitin (Fig. S3). Based on these results, we determined the optimal cefoxitin concentration to be 8 µg/ml.

**Detection of inducible AmpC**

AmpC β-lactamase activity before and after AmpC induction was determined through cefoxitin induction and cloxacillin inhibition in the 50 subset 1 isolates (Fig. S4 and Table S5). All 50 isolates produced a low level of AmpC before induction (Fig. S4A). After induction, the levels of AmpC increased in all isolates (Fig. S4B and S4C). The AmpC induction disk test results were positive for 98% of the subset 1 isolates. The isolate (KUEN095) that was not detected by the AmpC induction disk test had the lowest induced AmpC activity (8.8 nmol/ml/min).

**Supplementary Discussion**

**Inducible AmpC in CTX-S isolates**

Ninety-nine percent of the CTX-S isolates (61% of the study isolates) were resistant to cefoxitin and exhibited cefoxitin-cefotaxime antagonism, which indicates that these isolates were inducible strains (1). Antagonism refers to the development of resistance to weak inducers (i.e., cefotaxime) due to the increase in AmpC activity caused by the addition of a strong AmpC inducer (i.e., cefoxitin). This finding was confirmed by the results of the AmpC β-lactamase assay in a subset of 50 isolates, but the activity of induced AmpC was highly variable.

For AmpC induction, we used cefoxitin, a potent AmpC inducer used in disk-based detection tests and AmpC screening (2), but the optimal concentration for induction has not been investigated. We determined the optimal concentration to be 8 µg/ml, which is the lowest concentration that can adequately induce AmpC and avoid the antimicrobial (growth inhibitory) effects of cefoxitin. This concentration is identical to that used in the AmpC production screening test in the European Committee on Antimicrobial Susceptibility Testing (EUCAST) guidelines (3) and the breakpoint in the CLSI guidelines (4), allowing the growth of AmpC screening-positive and cefoxitin-resistant isolates. Further confirmatory studies using a wider range of ECC strains are required to validate this concentration.

**Supplementary Methods**

**Disk diffusion test supplemented with cefoxitin**

To determine the optimal cefoxitin concentration for AmpC induction, a disk diffusion test using cefotaxime (30 µg; Eiken Chemical) and ceftazidime (30 µg; Eiken Chemical) was performed according to the CLSI guidelines (4), modified to include cefoxitin supplementation with or without cloxacillin. Mueller‒Hinton agar (Becton Dickinson, New Jersey, USA) supplemented with cefoxitin (Toronto Research Chemicals, Toronto, Canada) at concentrations of 0, 4, 8, 16, 32 and 64 µg/ml was prepared on plates. To inhibit induced AmpC (restoration of zones of inhibition), Mueller‒Hinton agar plates were further supplemented with 16 or 128 µg/ml cloxacillin (Tokyo Kasei, Tokyo, Japan).

**Growth curve analysis**

Bacterial cultures were added to cation-adjusted Mueller‒Hinton broth (CAMHB; Eiken Chemical) to achieve a final concentration of 1×10^4^ CFU/ml. In addition, cefoxitin was added to the cultures at a final concentration of 8 µg/ml, and 100 µl of each culture was inoculated into 96-well plates and incubated at 35 °C. The optical density at 600 nm (OD_600_) was measured every 1 h for up to 24 h with a Nivo^TM^ S Multimode Plate Reader (PerkinElmer, Massachusetts, USA). Assays were performed in triplicate.

**Detection of inducible AmpC**

The AmpC induction disk test was performed using the double-disk synergy test using imipenem and cefotaxime disks as described by Dunne et al. (5). AmpC β-lactamase activity was assessed by nitrocefin hydrolysis. The bacterial suspensions were inoculated in 1.5 ml of CAMHB and incubated at 35 °C with shaking at 200 rpm until an OD_600_ of 0.2–0.3 was reached. Next, cefoxitin was added to the cultures at a final concentration of 8 µg/ml, and the cultures were further incubated for 0.5 h. The cultures were subsequently centrifuged at 2,300 × g at room temperature for 5 min and suspended in 50 mM sodium phosphate buffer (pH 7.0) to reach an OD_600_ of 0.2. One milliliter of bacterial suspension was washed with the same buffer and sonicated (ten 10 s pulses at 10 s intervals) on ice. The suspensions were subsequently centrifuged at 12,000 × g at 4 °C for 5 min, after which the supernatants were collected. Nitrocefin hydrolysis was measured with a Nivo^TM^ S Multimode Plate Reader. Samples containing 200 µM nitrocefin (Cayman Chemical, Michigan, USA) with or without 128 µg/ml cloxacillin were assayed every 1 min for 10 min at room temperature at an absorbance of 482 nm. *Klebsiella pneumoniae* ATCC 13883 was used as an AmpC-negative control, and ECC ATCC 13047 and ATCC 23355 were used as AmpC-positive controls. Assays were performed in triplicate.

**References**

1. Jeong SH, Song W, Park MJ, Kim JS, Kim HS, Bae IK, Lee KM. 2008. Boronic acid disk tests for identification of extended-spectrum beta-lactamase production in clinical isolates of Enterobacteriaceae producing chromosomal AmpC beta-lactamases. Int J Antimicrob Agents 31:467-71.

2. Jacoby GA. 2009. AmpC beta-lactamases. Clin Microbiol Rev 22:161-82.

3. EUCAST subcommittee for detection of resistance mechanisms and specific resistances of clinical and/or epidemiological importance. EUCAST guidelines for detection of resistance mechanisms and specific resistances of clinical and/or epidemiological importance. Version 2.0. July 2017. Available from <http://www.eucast.org/fileadmin/src/media/PDFs/EUCAST_files/Resistance_mechanisms/EUCAST_detection_of_resistance_mechanisms_170711.pdf>.

4. CLSI. 2022. Performance Standards for Antimicrobial Susceptibility Testing. 32nd ed. CLSI supplement M100. Wayne, PA: Clinical and Laboratory Standards Institute.

5. Dunne WM, Jr., Hardin DJ. 2005. Use of several inducer and substrate antibiotic combinations in a disk approximation assay format to screen for AmpC induction in patient isolates of Pseudomonas aeruginosa, Enterobacter spp., Citrobacter spp., and Serratia spp. J Clin Microbiol 43:5945-9.

**Table S1.** **Antimicrobial resistance genes other than β-lactamase genes carried by clinical *Enterobacter cloacae* complex isolates obtained from blood cultures at Kyoto University Hospital in Kyoto, Japan, 2002**–**2018.**

|  | N (%) | | |
| --- | --- | --- | --- |
| Variables | Total (n=194) | Cefotaxime-susceptible (n=120) | Cefotaxime-nonsusceptible (n=74) |
| *aac(2')-IIa* | 1 (1%) | 1 (1%) | 0 |
| *aac(3)-IId* | 1 (1%) | 0 | 1 (1%) |
| *aac(3)-IIg* | 3 (2%) | 0 | 3 (4%) |
| *aac(6')-31* | 2 (1%) | 0 | 2 (3%) |
| *aac(6')-Iaj*^a^ | 4 (2%) | 0 | 4 (5%) |
| *aac(6')-Ib3* | 1 (1%) | 1 (1%) | 0 |
| *aac(6')-Ib4* | 1 (1%) | 0 | 1 (1%) |
| *aac(6')-IIc* | 3 (2%) | 0 | 3 (4%) |
| *aac(6')-Il* | 1 (1%) | 0 | 1 (1%) |
| *aadA1*^a^ | 4 (2%) | 0 | 4 (5%) |
| *aadA2* | 9 (5%) | 4 (3%) | 5 (7%) |
| *ant(2'')-Ia* | 2 (1%) | 1 (1%) | 1 (1%) |
| *aph(3'')-Ib* | 26 (13%) | 17 (14%) | 9 (12%) |
| *aph(6)-Id* | 26 (13%) | 17 (14%) | 9 (12%) |
| *arr* | 3 (2%) | 0 | 3 (4%) |
| *catA2* | 7 (4%) | 3 (3%) | 4 (5%) |
| *catB6* | 1 (1%) | 0 | 1 (1%) |
| *cmlA1* | 3 (2%) | 0 | 3 (4%) |
| *dfrA12* | 1 (1%) | 0 | 1 (1%) |
| *dfrA14* | 14 (7%) | 11 (9%) | 3 (4%) |
| *dfrA15*^a^ | 9 (5%) | 0 (0%) | 9 (12%) |
| *dfrA16* | 2 (1%) | 2 (2%) | 0 |
| *dfrA19*^a^ | 5 (3%) | 0 | 5 (7%) |
| *dfrA8* | 2 (1%) | 0 | 2 (3%) |
| *ere(A)* | 4 (2%) | 1 (1%) | 3 (4%) |
| *fosA*^a^ | 165 (85%) | 97 (81%) | 68 (92%) |
| *fosA2* | 10 (5%) | 7 (6%) | 3 (4%) |
| *fosA7* | 2 (1%) | 2 (2%) | 0 |
| *mcr-10* | 2 (1%) | 1 (1%) | 1 (1%) |
| *mcr-10.1* | 12 (6%) | 9 (8%) | 3 (4%) |
| *mph(A)* | 1 (1%) | 0 | 1 (1%) |
| *oqxA* | 190 (98%) | 117 (98%) | 73 (99%) |
| *oqxB* | 191 (98%) | 117 (98%) | 74 (100%) |
| *qacE* | 1 (1%) | 0 | 1 (1%) |
| *qacEΔ1*^a^ | 19 (10%) | 4 (3%) | 15 (20%) |
| *qnrA1* | 2 (1%) | 1 (1%) | 1 (1%) |
| *qnrB4* | 1 (1%) | 0 | 1 (1%) |
| *qnrE* | 12 (6%) | 7 (6%) | 5 (7%) |
| *qnrE3* | 1 (1%) | 1 (1%) | 0 |
| *qnrS1* | 5 (3%) | 2 (2%) | 3 (4%) |
| *sul1*^a^ | 20 (10%) | 4 (3%) | 16 (22%) |
| *sul2* | 16 (8%) | 11 (9%) | 5 (7%) |
| *tet(A)* | 2 (1%) | 2 (2%) | 0 |
| *tet(D)* | 7 (4%) | 4 (3%) | 3 (4%) |

^a^ P value <0.05 for cefotaxime-susceptible vs. cefotaxime-nonsusceptible isolates.

**Table S2. Comparison of major species, sequence types, antimicrobial non-susceptibilities, and β-lactamase genes of *Enterobacter cloacae* complex isolates between the isolation years 2002–10 and 2011–2018.**

|  | N (%) | |
| --- | --- | --- |
| Variables | 2002–10 (n=77) | 2011–2018 (n=117) |
| Species |  |  |
| *Enterobacter xiangfangensis* | 26 (34%) | 43 (37%) |
| *Enterobacter ludwigii* | 8 (10%) | 17 (15%) |
| *Enterobacter kobei* | 12 (16%) | 12 (10%) |
| *Enterobacter asburiae* | 11 (14%) | 12 (10%) |
| *Enterobacter hoffmannii* | 6 (8%) | 7 (6%) |
| *Enterobacter cloacae* | 6 (8%) | 3 (3%) |
| *Enterobacter roggenkampii* | 4 (5%) | 4 (3%) |
| *Enterobacter bugandensis* | 1 (1%) | 6 (5%) |
| Sequence type |  |  |
| ST78 | 5 (6%) | 6 (5%) |
| ST93 | 4 (5%) | 2 (2%) |
| ST116 | 1 (1%) | 5 (4%) |
| ST252 | 4 (5%) | 2 (2%) |
| ST45 | 3 (4%) | 2 (2%) |
| ST20 | 3 (4%) | 1 (1%) |
| ST32^a^ | 4 (5%) | 0 (0%) |
| ST50 | 1 (1%) | 3 (3%) |
| Antimicrobial non-susceptibility |  |  |
| Cefotaxime | 28 (36%) | 46 (39%) |
| Ceftazidime | 21 (27%) | 34 (29%) |
| Cefepime | 9 (12%) | 5 (4%) |
| Piperacillin | 28 (36%) | 43 (37%) |
| Piperacillin-tazobactam | 17 (22%) | 26 (22%) |
| Aztreonam | 20 (26%) | 28 (24%) |
| Ciprofloxacin | 17 (22%) | 14 (12%) |
| Levofloxacin | 15 (19%) | 12 (10%) |
| Gentamicin | 2 (3%) | 2 (2%) |
| Tobramycin^a^ | 7 (9%) | 1 (1%) |
| Amikacin | 3 (4%) | 0 (0%) |
| Minocycline | 9 (12%) | 10 (9%) |
| Sulfamethoxazole-trimethoprim | 16 (21%) | 15 (13%) |
| Colistin | 29 (38%) | 38 (32%) |
| β-Lactamase gene |  |  |
| *bla*_ACT_ | 66 (86%) | 109 (93%) |
| *bla*_CMH_ | 7 (9%) | 3 (3%) |
| *bla*_MIR_ | 4 (5%) | 4 (3%) |
| *bla*_CTX-M-3_^a^ | 7 (9%) | 2 (2%) |
| *bla*_SHV-12_ | 2 (3%) | 1 (1%) |
| *bla*_LAP-2_ | 1 (1%) | 2 (2%) |
| *bla*_TEM-1_ | 7 (9%) | 9 (8%) |

^a^ P value of <0.05 for 2002–10 vs. 2011–2018.

**Table S3. Inhibitory zone diameter of cefotaxime disks on Mueller-Hinton agar with no agent, 128 µg/ml cloxacillin, 8 μg/ml cefoxitin, or 8 µg/ml cefoxitin plus cloxacillin.**

|  | Inhibitory zone diameter of cefotaxime disk (mm) | | | |
| --- | --- | --- | --- | --- |
| Group^a^, isolate | No agent | Cloxacillin^b^ | 8 μg/ml cefoxitin | 8 µg/ml cefoxitin  plus cloxacillin^b^ |
| Group A |  |  |  |  |
| KUEN22 | 29 | 32 | 21 | 29 |
| KUEN27 | 28 | 29 | 23 | 30 |
| KUEN28 | 30 | 31 | 22 | 29 |
| Group B |  |  |  |  |
| KUEN111 | 32 | 31 | 16 | 30 |
| KUEN187 | 32 | 34 | 20 | 34 |
| KUEN193 | 34 | 35 | 21 | 33 |
| Group C |  |  |  |  |
| KUEN114 | 32 | 33 | 21 | 34 |
| KUEN125 | 31 | 31 | 22 | 31 |
| KUEN177 | 31 | 34 | 22 | 34 |

^a^ The median diameter and diameter change of the cefotaxime disk among subset 2 isolates were 31 mm and 9 mm, respectively (Dataset 1). Group A showed a smaller diameter and/or smaller diameter change, group B showed a moderate diameter and/or moderate change, and group C showed a larger diameter and/or larger change.

^b^ 16 µg/ml cloxacillin was used for group A isolates, and 128 µg/ml cloxacillin was used for group B and C isolates.

**Table S4. Inhibitory zone diameter of ceftazidime disks on Mueller-Hinton agar with no agent, 128 µg/ml cloxacillin, 8 μg/ml cefoxitin, or 8 µg/ml cefoxitin plus cloxacillin.**

|  | Inhibitory zone diameter of ceftazidime disk (mm) | | | |
| --- | --- | --- | --- | --- |
| Group^a^, isolate | No agent | Cloxacillin^b^ | 8 μg/ml cefoxitin | 8 µg/ml cefoxitin  plus cloxacillin^b^ |
| Group A |  |  |  |  |
| KUEN22 | 27 | 28 | 24 | 28 |
| KUEN27 | 25 | 27 | 23 | 27 |
| KUEN28 | 27 | 27 | 22 | 28 |
| Group B |  |  |  |  |
| KUEN111 | 30 | 27 | 18 | 27 |
| KUEN187 | 28 | 31 | 18 | 31 |
| KUEN193 | 30 | 29 | 20 | 28 |
| Group C |  |  |  |  |
| KUEN114 | 28 | 29 | 20 | 30 |
| KUEN125 | 28 | 27 | 22 | 29 |
| KUEN177 | 29 | 29 | 20 | 28 |

^a^ The median diameter and diameter change of the ceftazidime disk among subset 2 isolates were 28 mm and 8.5 mm, respectively (Dataset 1). Group A showed a smaller diameter and/or smaller diameter change, group B showed a moderate diameter and/or moderate change, and group C showed a larger diameter and/or larger change.

^b^ 16 µg/ml cloxacillin was used for group A isolates, and 128 µg/ml cloxacillin was used for group B and C isolates.

**Table S5. β-Lactamase activities of 50 isolates** **before and after induction of AmpC.**

|  | Mean β-lactamase activity (nmol/ml/min) | | | | Induced AmpC β-lactamase activity^a^ | |
| --- | --- | --- | --- | --- | --- | --- |
|  | Not induced | | Induced | | Value (nmol/ml/min) | Fold change |
| Isolate | Cloxacillin (-) | Cloxacillin (+) | Cloxacillin (-) | Cloxacillin (+) |  |  |
| KUEN001 | 0.76 | 0 | 24 | 0 | 23 | 32 |
| KUEN002 | 1.5 | 0 | 99 | 0 | 98 | 66 |
| KUEN003 | 4.5 | 0.90 | 63 | 0 | 59 | 18 |
| KUEN021 | 0.29 | 0 | 86 | 0 | 86 | 300 |
| KUEN022 | 0.067 | 0 | 37 | 0 | 37 | 550 |
| KUEN023 | 0.67 | 0 | 24 | 0.67 | 23 | 35 |
| KUEN026 | 1.2 | 0.067 | 120 | 0 | 120 | 110 |
| KUEN027 | 0.43 | 0 | 57 | 0 | 57 | 130 |
| KUEN028 | 0.19 | 0 | 75 | 0 | 75 | 390 |
| KUEN042 | 0.25 | 0 | 96 | 0 | 96 | 380 |
| KUEN043 | 0.30 | 0 | 33 | 0 | 33 | 110 |
| KUEN044 | 7.8 | 2.7 | 120 | 2.7 | 110 | 23 |
| KUEN048 | 1.1 | 0.067 | 42 | 0 | 41 | 41 |
| KUEN049 | 0.80 | 0 | 68 | 0 | 67 | 85 |
| KUEN054 | 0.57 | 0.067 | 55 | 0 | 55 | 110 |
| KUEN055 | 1.1 | 0 | 23 | 0 | 22 | 21 |
| KUEN060 | 1.5 | 0 | 47 | 0 | 46 | 31 |
| KUEN061 | 1.3 | 0.13 | 130 | 0 | 130 | 110 |
| KUEN062 | 0.71 | 0 | 89 | 0 | 88 | 130 |
| KUEN063 | 1.4 | 0 | 80 | 0 | 79 | 57 |
| KUEN064 | 0.44 | 0 | 24 | 0.67 | 23 | 53 |
| KUEN076 | 1.0 | 0.027 | 31 | 0.13 | 30 | 32 |
| KUEN077 | 0.31 | 0 | 50 | 0 | 50 | 160 |
| KUEN078 | 0.49 | 0.089 | 130 | 0 | 130 | 320 |
| KUEN094 | 5.9 | 1.7 | 60 | 1.3 | 55 | 14 |
| KUEN095 | 0.29 | 0 | 9.2 | 0.067 | 8.8 | 32 |
| KUEN096 | 0.76 | 0.089 | 42 | 0 | 41 | 63 |
| KUEN109 | 0.9 | 0 | 37 | 0 | 36 | 41 |
| KUEN111 | 0.53 | 0.022 | 200 | 2.0 | 200 | 390 |
| KUEN114 | 1.2 | 0.089 | 30 | 0 | 29 | 27 |
| KUEN125 | 0.77 | 0 | 110 | 0.67 | 110 | 140 |
| KUEN126 | 0.63 | 0.033 | 34 | 0 | 33 | 57 |
| KUEN127 | 0.75 | 0 | 68 | 2.0 | 65 | 88 |
| KUEN141 | 9.4 | 0 | 41 | 0 | 32 | 4 |
| KUEN142 | 1.8 | 0 | 99 | 0 | 97 | 55 |
| KUEN158 | 1.4 | 0 | 28 | 0 | 27 | 20 |
| KUEN159 | 0.43 | 0 | 63 | 0.67 | 62 | 150 |
| KUEN163 | 0.77 | 0 | 17 | 0.67 | 16 | 21 |
| KUEN164 | 1.2 | 0 | 79 | 0 | 78 | 66 |
| KUEN167 | 0.76 | 0 | 61 | 0 | 60 | 80 |
| KUEN173 | 1.1 | 0 | 250 | 0 | 250 | 230 |
| KUEN174 | 0.76 | 0 | 31 | 0 | 30 | 41 |
| KUEN177 | 1.2 | 0 | 25 | 0 | 24 | 21 |
| KUEN185 | 0.42 | 0 | 13 | 1.3 | 11 | 28 |
| KUEN187 | 1.5 | 0 | 80 | 1.3 | 77 | 53 |
| KUEN191 | 0.93 | 0.044 | 27 | 0 | 26 | 31 |
| KUEN192 | 1.3 | 0.053 | 89 | 0 | 88 | 71 |
| KUEN193 | 1.4 | 0 | 95 | 0 | 94 | 68 |
| KUEN199 | 0.87 | 0 | 35 | 0 | 34 | 40 |
| KUEN200 | 0.36 | 0 | 20 | 0 | 20 | 56 |
| *Klebsiella pneumoniae* ATCC 13383^b,c^ | 0.49 | 0.44 | 0.53 | 0.49 | -0.01 | 0.8 |
| *Escherichia coli* ATCC 25922^b^ | 0 | 0 | 0.40 | 0 | 0.4 | NC |
| *Enterobacter cloacae*  ATCC 13047^d^ | 7.0 | 0 | 224 | 0 | 217 | 32 |
| *Enterobacter cloacae*  ATCC 23355 ^d,e^ | 0.87 | 0 | 7.1 | 0 | 6.2 | 8.2 |

NC, not calculated.

^a^ AmpC β-lactamase activity of the non-induced or induced conditions was calculated by the β-lactamase activity without cloxacillin minus that with cloxacillin.

^b^ AmpC-negative reference strain.

^c^ ATCC 13383 carries only a broad-spectrum β-lactamase SHV-1 and exhibited a β-lactamase activity of 0.49 nmol/ml/min and an AmpC β-lactamase activity of 0 (an actual value of -0.01, considered within the margin of error).

^d^ Inducible AmpC-positive reference strain.

^e^ ATCC 23355 exhibited an 8.2-fold increase in AmpC β-lactamase activity upon induction, which was consistent with a previous study reporting a 12-fold increase (under different culture and induction conditions; Ishii Y, Ichikawa M, Yamaguchi K, Takano K, Inoue M. 1991. Localization of cephalosporinase in *Enterobacter cloacae* by immunocytochemical examination. J Antibiot (Tokyo) 44:1088-95).

**Table S6.** ***ampC*-derepressed mutants obtained from 50 cefotaxime-susceptible *Enterobacter cloacae* complex isolates.**

|  | Antimicrobial agents used for cultures, n (%) | | |
| --- | --- | --- | --- |
| Variables | None | Cefoxitin | Cefoxitin and cefotaxime |
| Number of isolates that produced mutants | 44 (88%) | 45 (90%) | 33 (66%) |
| Mean mutation frequency | 4.5×10^-6^ | 6.1×10^-6^ | 3.2×10^-3^ |
| Mutation frequency >0.1 | 0 | 0 | 9 (18%) |
| Mean mutation ratio^b^ | Reference | 1.6 | 5.7×10^2^ |
| Mutation ratio^b^, >10 | Reference | 8 (16%) | 25 (50%) |
| Mutation ratio^b^, 0.1–10 | Reference | 31 (62%) | 7 (14%) |
| Mutation ratio^b^, <0.1 | Reference | 3 (6%) | 1 (2%) |
| Mutants obtained only in cultures with the antimicrobials |  | 3 (6%) | 0 |
| Mutants obtained only in cultures without the antimicrobials |  | 2 (4%) | 11 (22%) |
| Mutants not obtained in cultures with or without the antimicrobials |  | 3 (6%) | 6^a^ (12%) |

^a^ Two isolates did not grow with these antimicrobial agents.

^b^ Mutation ratios were calculated by dividing *ampC*-derepressed mutation frequencies in cultures with cefoxitin or cefoxitin and cefotaxime by those without antimicrobials.


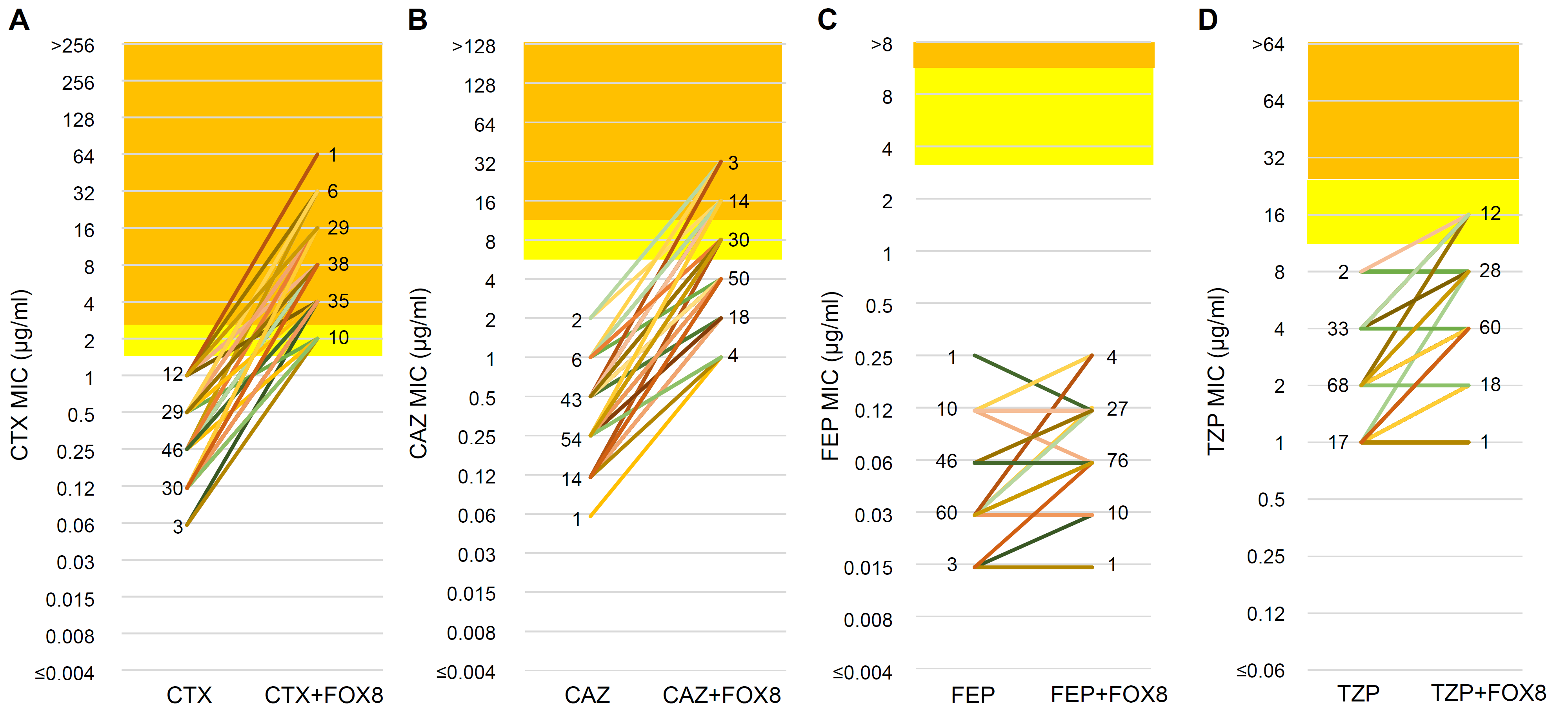


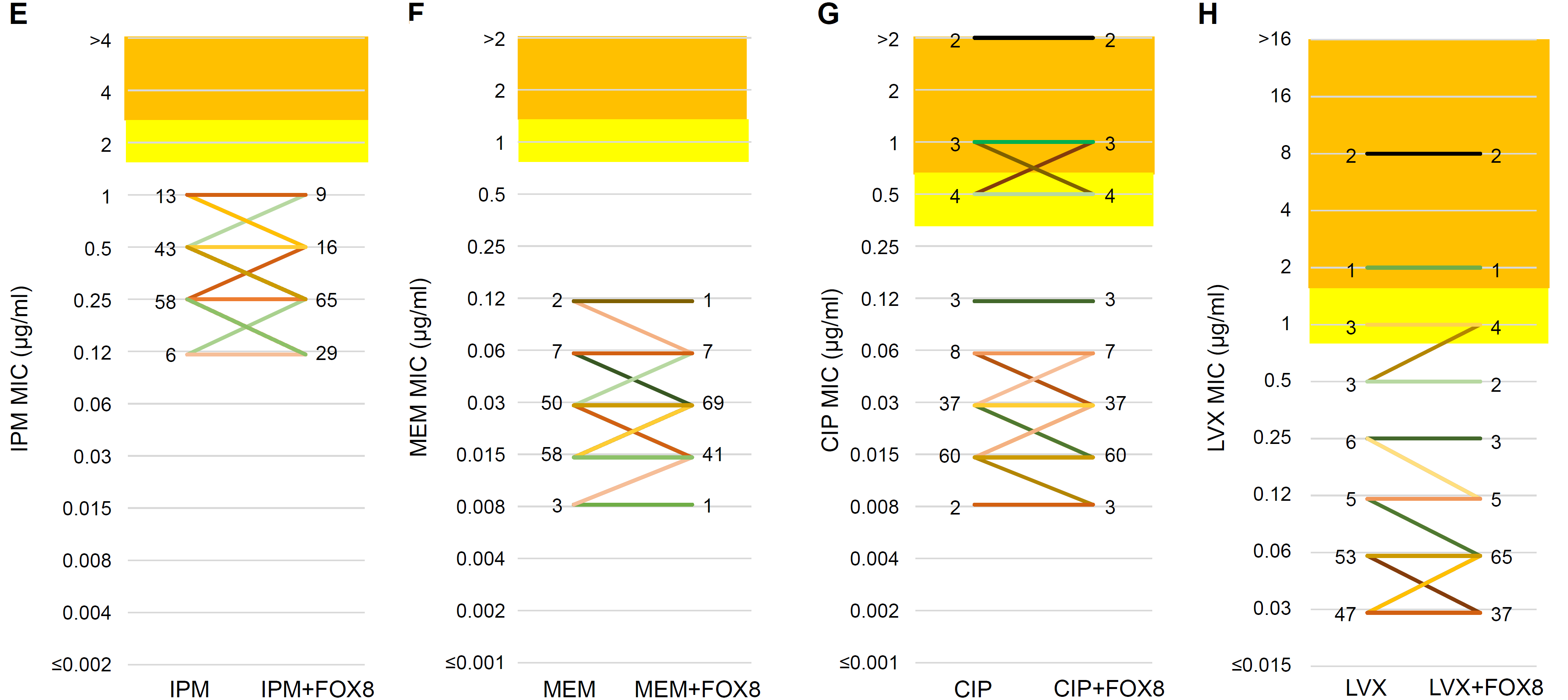


**Fig. S1. Comparison of the MICs of cefotaxime (A), ceftazidime (B), cefepime (C), piperacillin/tazobactam (D), imipenem (E), meropenem (F), ciprofloxacin (G), and levofloxacin (H) with or without 8 µg/ml cefoxitin (cefoxitin antagonism test) among 119 cefotaxime-susceptible and cefoxitin-resistant isolates.** The number of wild-type or mutant isolates with each MIC value is shown next to the connecting lines. The susceptible, intermediate or susceptible dose-dependent, and resistant categories are indicated by white, yellow, and orange backgrounds, respectively.

**
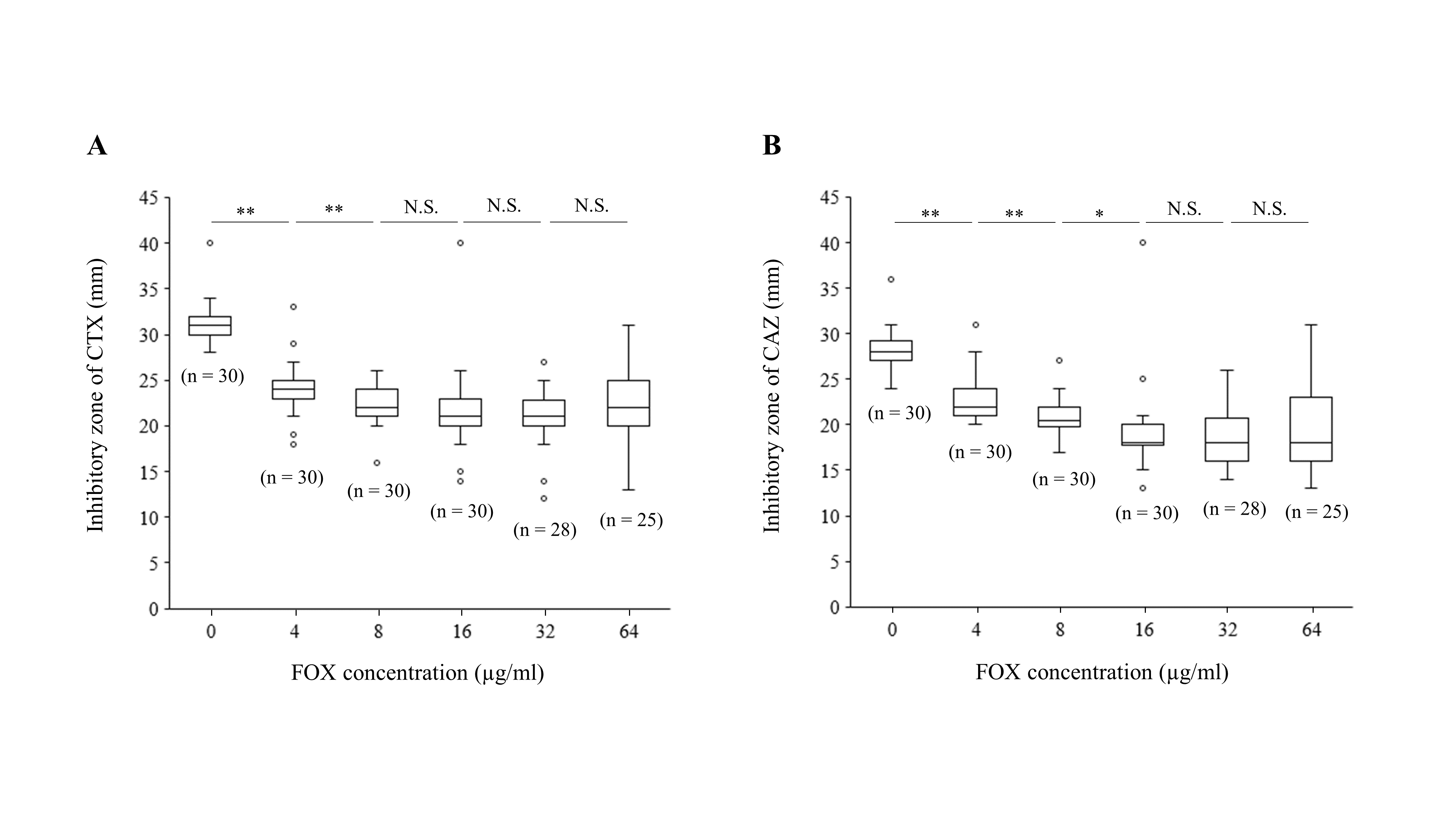
**

**Fig. S2. Inhibitory zone diameters of (A) cefotaxime and (B) ceftazidime discs on Mueller-Hinton agar supplemented with different cefoxitin concentrations among the 30 subset 2 *Enterobacter cloacae* complex isolates.** * *P* < 0.01; ** *P* < 0.001; N.S., not significant; Mann–Whitney U test. Bacterial growth was not observed for 2 isolates at 32 µg/ml cefoxitin and for 5 isolates at 64 µg/ml cefoxitin.

**
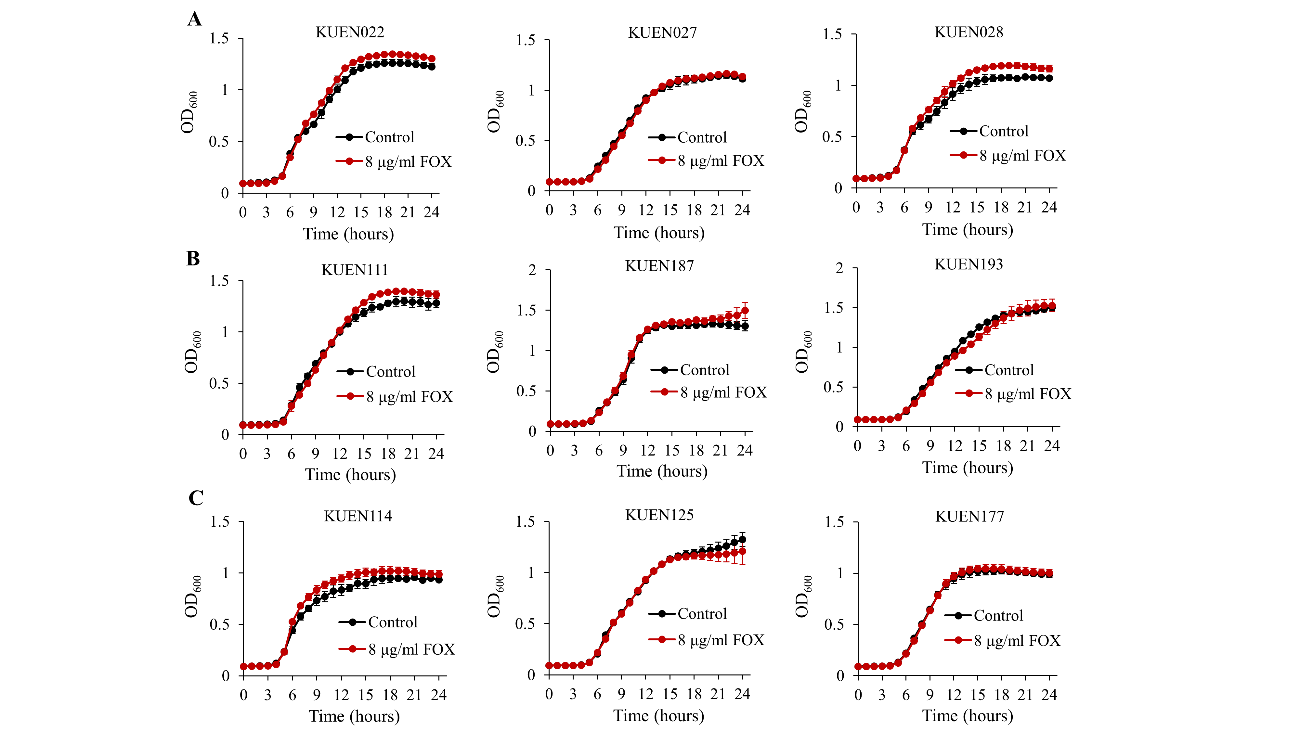
**

**Fig. S3. Growth curve analysis with and without 8 µg/ml cefoxitin for the 9 representative isolates from the 3 groups showing different cefotaxime/ceftazidime inhibitory zone diameters.** Group A includes isolates with the smallest diameter and/or the smallest diameter change. Group B includes isolates with a moderate diameter and/or moderate change. Group C includes isolates with the largest diameter and/or the largest change. The mean ± standard deviation of OD_600_ values are shown at an interval of 1 h.


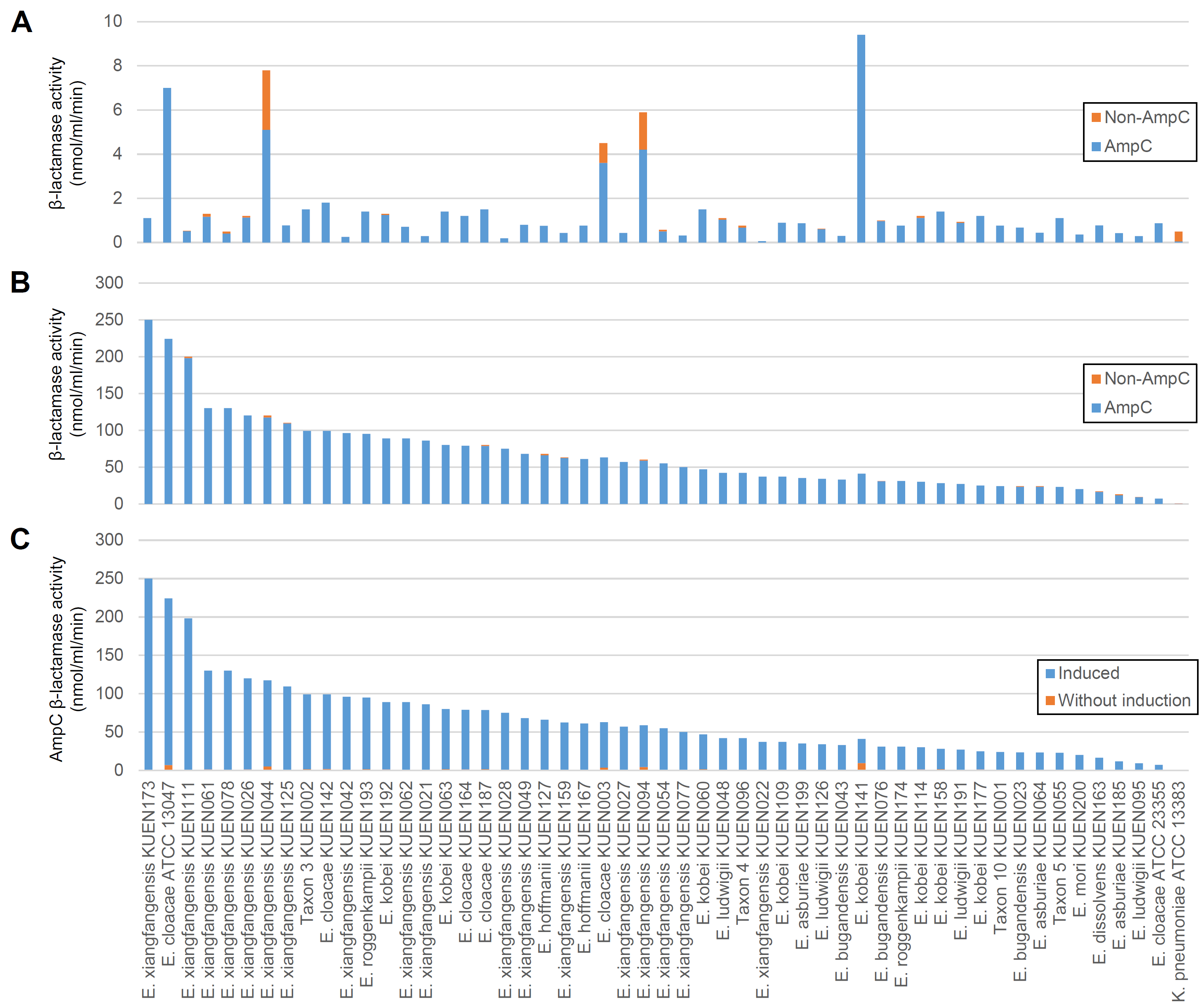


**Fig.** **S4. β-lactamase activities of 50 cefotaxime-susceptible *Enterobacter cloacae* complex isolates.** Panel A shows β-lactamase activity before induction. All the isolates produced a low level of AmpC (median, 0.785 nmol/ml/min; range, 0.067–9.4). Non-AmpC β-lactamase activity was detected in three isolates, all of which carried TEM-1 penicillinase. Panel B shows β-lactamase activities measured with cefoxitin induction, and Panel C shows AmpC β-lactamase activities with and without cefoxitin induction. All the isolates produced AmpC at higher levels after induction (median, 55 nmol/ml/min; range, 8.8–250; median fold change, 57; range of fold changes, 4.4–550). Note that the Y-axis scale is different between panel A and panels B or C. *K. pneumoniae* ATCC 13883 was used as an AmpC-negative control, and ECC ATCC 13047 and ATCC 23355 were used as AmpC-positive controls.
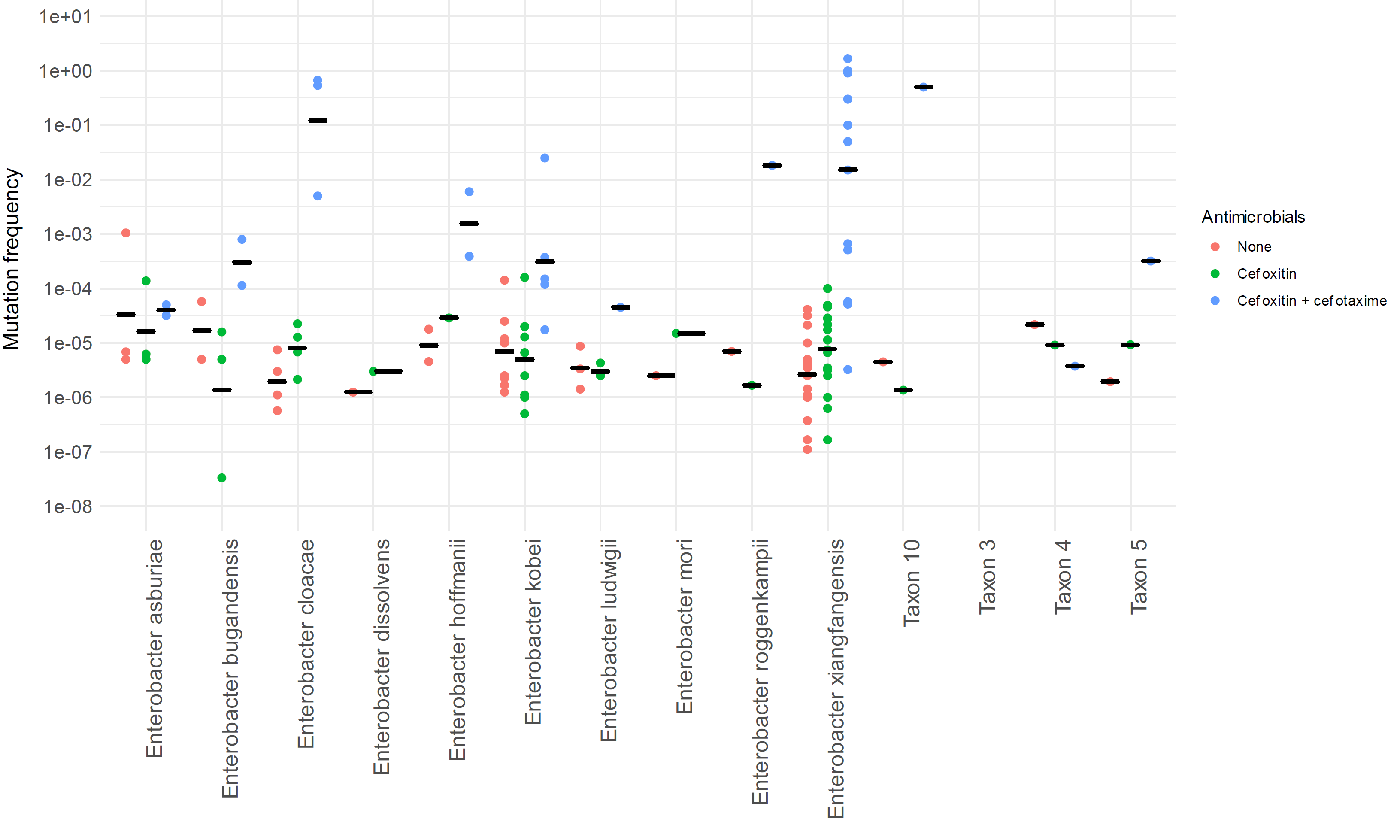


**Fig. S5. *ampC*-derepressed mutation frequencies among 50 cefotaxime-susceptible *Enterobacter cloacae* complex isolates during broth microdilution antimicrobial susceptibility testing, according to species.** The blue bars indicate the geometric means. Two isolates (*E. roggenkampii*, n=1; taxon 3, n=1) did not grow in cultures with cefoxitin and cefotaxime.


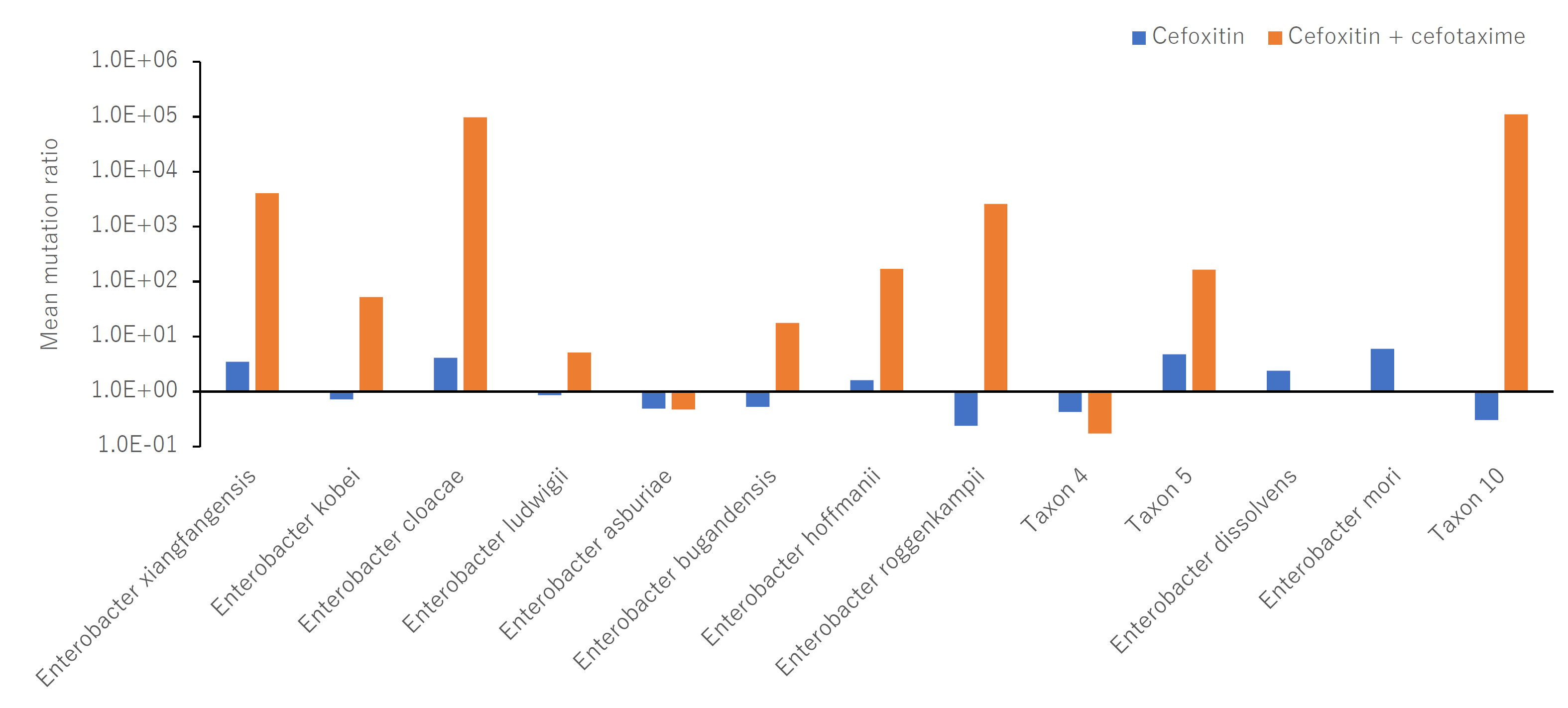


**Fig. S6. Mutation ratios (*ampC*-derepressed mutation frequencies in cultures with cefoxitin or cefoxitin and cefotaxime divided by those without antimicrobials)** **among 50 cefotaxime-susceptible *Enterobacter cloacae* complex isolates.** Blue bars indicate the geometric mean. The mutation ratios of cefoxitin and cefotaxime for *E. dissolvens* and *E. mori*, and that for taxon 3 are not shown because the mutation frequencies could not be calculated due to the absence of mutants or inability to grow in cultures with the antimicrobial agents.
